# High specificity of engineered T cells with third generation CAR (CD28-4-1BB-CD3-ζ) based on biotin-bound monomeric streptavidin for potential tumor immunotherapy

**DOI:** 10.3389/fimmu.2024.1448752

**Published:** 2024-09-19

**Authors:** Jorge Gallego-Valle, Verónica Astrid Pérez-Fernández, Jesús Rosales-Magallares, Sergio Gil-Manso, María Castellá, Europa Azucena Gonzalez-Navarro, Rafael Correa-Rocha, Manel Juan, Marjorie Pion

**Affiliations:** ^1^ Group of Advanced Immuno-Regulation (GIRA), Gregorio Marañon Health Research Institute Instituto de Investigación Sanitaria Gregorio Marañón (IiSGM), Hospital General Gregorio Marañon, Madrid, Spain; ^2^ Immune-Regulation Laboratory (LIR), Instituto de Investigación Sanitaria Gregorio Marañón (IiSGM), Hospital General Gregorio Marañon, Madrid, Spain; ^3^ Immunology Service, Centre for Biomedical Diagnosis (CDB), Hospital Clínic de Barcelona (HCB), Joint Platform for Immunotherapy of Hospital Sant Joan de Deu, Barcelona, Spain

**Keywords:** engineered cells, immunosuppression, Treg, Chimeric Antigen Receptor, streptavidin-based CAR

## Abstract

**Introduction:**

Immunotherapy has revolutionized cancer treatment, and Chimeric Antigen Receptor T cell therapy (CAR-T) is a groundbreaking approach. Traditional second-generation CAR-T therapies have achieved remarkable success in hematological malignancies, but there is still room for improvement, particularly in developing new targeting strategies. To address this limitation, engineering T cells with multi-target universal CARs (UniCARs) based on monomeric streptavidin has emerged as a versatile approach in the field of anti-tumor immunotherapy. However, no studies have been conducted on the importance of the intracellular signaling domains of such CARs and their impact on efficiency and specificity

**Method:**

Here, we developed second-generation and third-generation UniCARs based on an extracellular domain comprising an affinity-enhanced monomeric streptavidin, in addition to CD28 and 4-1BB co-stimulatory intracellular domains. These UniCAR structures rely on a biotinylated intermediary, such as an antibody, for recognizing target antigens. In co-culture assays, we performed a functional comparison between the third-generation UniCAR construct and two second-generation UniCAR variants, each incorporating either the CD28 or 4-1BB as co-stimulatory domain

**Results:**

We observed that components in culture media could inhibit the binding of biotinylated antibodies to monomeric streptavidin-CARs, potentially compromising their efficacy. Furthermore, third-generation UniCAR-T cells showed robust cytolytic activity against cancer cell lines upon exposure to specific biotinylated antibodies like anti-CD19 and anti-CD20, underscoring their capability for multi-targeting. Importantly, when assessing engineered UniCAR-T cell activation upon encountering their target cells, third-generation UniCAR-T cells exhibited significantly enhanced specificity compared to second-generation CAR-T cells

**Discussion:**

First, optimizing culture conditions would be essential before deploying UniCAR-T cells clinically. Moreover, we propose that third-generation UniCAR-T cells are excellent candidates for preclinical research due to their high specificity and multi-target anti-tumor cytotoxicity

## 1 Introduction

Malignant tumors pose a significant global health concern due to their increasing annual incidence, some coupled with a still insufficient response rate to available therapies for achieving long-term remission (1). One of the most successful strategies that have emerged in recent years, particularly in the treatment of hematological malignancies, is adoptive immunotherapy based on Chimeric Antigen Receptor T (CAR-T) cells (2–4). Engineered CAR-T cells recognize and target tumor-associated antigens (Ags) on cancer cells.

Conventional CARs are synthetic proteins formed by an extracellular domain that binds to a specific Ag on the surface of cancer cells, a hinge domain, a transmembrane domain and intracellular signaling domains (5). The CAR extracellular domain is often a single-chain variable fragment (ScFv) that maintains the original affinity and specificity for an Ag, such as CD19 on malignant B cells (6, 7). The intracellular region is the one that provides the necessary signals for CAR-bearing T cell activation. Depending on the domains of the intracellular region, several CAR generations have been developed. First generation CAR is solely composed of the CD3-ζ chain, which is responsible for the primary T cell activation signaling (8). To mimic the physiological T cell activation, the intracellular domain of second-generation CARs combined the CD3-ζ chain with an additional co-stimulation signal provided by CD28, 4-1BB, CD27, OX40 or ICOS proteins, among others (9). The third generation CAR is composed of the CD3-ζ chain next to two co-stimulatory domains. This combination can induce a higher rate of proliferation and greater long-term survival *in vivo*, while causing less cellular exhaustion in engineered T cells compared to second-generation CARs (10).

Although CAR-T therapy has shown remarkable success, some drawbacks still limit its global implementation. Significant toxicities, such as cytokine release syndrome and neurotoxicity, can be life-threatening in severe cases. Additionally, CAR-T therapy is currently only feasible for certain types of cancer and has shown limited efficiency against solid tumors, restricting its availability to a broader patient population (2, 11–13). Addressing these challenges requires further research to optimize this technology and expand its applicability to a wider range of cancer patients.

To overcome some of the limitations outlined for CAR-T cell technology, various approaches have been developed. Universal CARs (UniCARs) stem from the modification of conventional CARs, utilizing small molecules as extracellular intermediaries that recognize the desired target antigen (14). The main advantage of UniCARs, through this intermediary approach, is their ability to use a single CAR construct against multiple therapeutic targets by simply changing the intermediary, making it a flexible tool. This approach has the potential to reduce treatment costs and enhance scalability (15). Moreover, because UniCARs depend on an intermediary to achieve target recognition, this technology offers the possibility to modulate CAR-mediated T cell activation and function by regulating the availability of the intermediary. This approach holds potential for mitigating conventional CAR-related toxicity (16).

One of the most promising approaches for UniCARs is based on the interaction between biotin and biotin-binding proteins (BBP) (15). Lohmueller et al. developed two second-generation UniCARs with extracellular domains containing monomeric streptavidin 2 (mSA2)-UniCAR (17). This mSA2-UniCAR structure addresses both the immunogenicity and non-specificity limitations observed in other BBP-based UniCARs, while retaining remarkable biotin affinity (Kd= 5.5 x 10^9^at 37°C) (18). In our study, we tested the extracellular domain mSA2-UniCAR generated by Lohmueller et al. (17) in a third-generation construct combining the co-stimulatory domains 4-1BB and CD28. The anti-tumor functionality of this combination was compared against academic conventional anti-CD19 CAR (CAR19; ARI-0001 CAR-T) (19–21) and second-generation mSA2-UniCARs based on 4-1BB or CD28. We demonstrated that the combination of these co-stimulatory domains positively influenced the specificity of mSA2-UniCAR-T cells. Third-generation mSA2-UniCAR-28-BB-T cells exhibited greater specificity compared to both second-generation mSA2-UniCAR-T cells, while retaining the elevated anti-tumor activity characteristic of conventional CAR19-T cells. This suggests that the third-generation mSA2-UniCAR-28-BB-T cells are a promising therapeutic approach, combining high specificity and potent anti-tumor activity, thus addressing some limitations of previous CAR-T cell technologies.

## 2 Materials and methods

### 2.1 Primary human T cell culture and lentiviral transduction

Peripheral Blood Mononuclear Cells (PBMC) were isolated from deidentified human Buffy Coats from healthy volunteer donors obtained from the Madrid Transfusion Center. PBMC were isolated using Ficoll centrifugation and cultured at 37°C and 5% of CO2 in X-vivo15 (Lonza, Walkersville, MD, USA) supplemented with 5% of human AB serum (ABS, Sigma-Aldrich, St. Louis, MO, USA) and 300 U/ml IL-2 (ImmunoTools, Friesoythe, Germany). PBMCs were stimulated and expanded using αCD3/αCD28 Dynabeads Human T-Activator (Invitrogen, Waltham, MA, USA) for 48 hours; then, activated cells were transduced with lentivectors (100 to 300 ng of p24gag per 1.5 million cells). Cells were then washed and cultured for 3 additional days for their expansion. Every UniCAR+ cells (2nd or 3rd generation) expressing Green Fluorescent Protein (eGFP), were flow-sorted by MACSQuant^®^Tyto^®^(Miltenyi Biotec, Bergisch Gladbach, Germany) and then cultured at 37°C and 5% of CO2 in RPMI 1640 + 10% fetal bovine serum (FBS; Biowest, Nuaillé, France) and 1% antibiotic mix (125 µg/ml of ampicillin, 125 µg/ml of cloxacillin and 40 µg/ml of gentamicin, Merck Life Science, Madrid, Spain), supplemented with 300 U/ml IL-2. 24 hours after sorting, cells were analyzed for viability and purity by flow cytometry.

### 2.2 Cell line culture

Human tumor cell lines Jurkat Clone E6–1 (TIB-152, RRID: CVCL_0367) and Raji (CCL-86, RRID: CVCL_0511) were obtained from Reagent Program (NIH, Bethesda, MD, USA); and K562 (CCL-243, RRID: CVCL_0004) were obtained from European Collection of Authenticated Cell Cultures (Salisbury, UK). These cell lines were cultured at 37°C and 5% of CO2 in RPMI 1640 (Biochrome, Holliston, MA, USA) medium supplemented with 10% FBS and 1% antibiotic mix. UniCAR-28-BB+ Jurkat cells were generated by transducing Jurkat cells with the third-generation UniCAR-28-BB expressing-lentivirus. Transduced UniCAR-28-BB+ Jurkat were enriched by sorting cells for positive eGFP expression. CD19+ K562 cells (K562-CD19) were generated by transducing K562 cells with full-length CD19 expressing-lentivirus (VectorBuilder GmbH, Neu-Isenburg, Germany). Transduced K562-CD19 were enriched by sorting CD19+ cells. Human embryonic kidney (HEK)-293T cells (American Type Culture Collection, ATCC; RRID: CVCL_0063, LGC Standards S.L.U., Barcelona, Spain) were cultured at 37°C and 5% of CO2 in high glucose-DMEM (Gibco, ThermoFisher, Waltham, MA, USA) supplemented with 10% FBS, and 1% antibiotic mix.

### 2.3 Lentiviral vector construction and virus production

**Figure 1:**
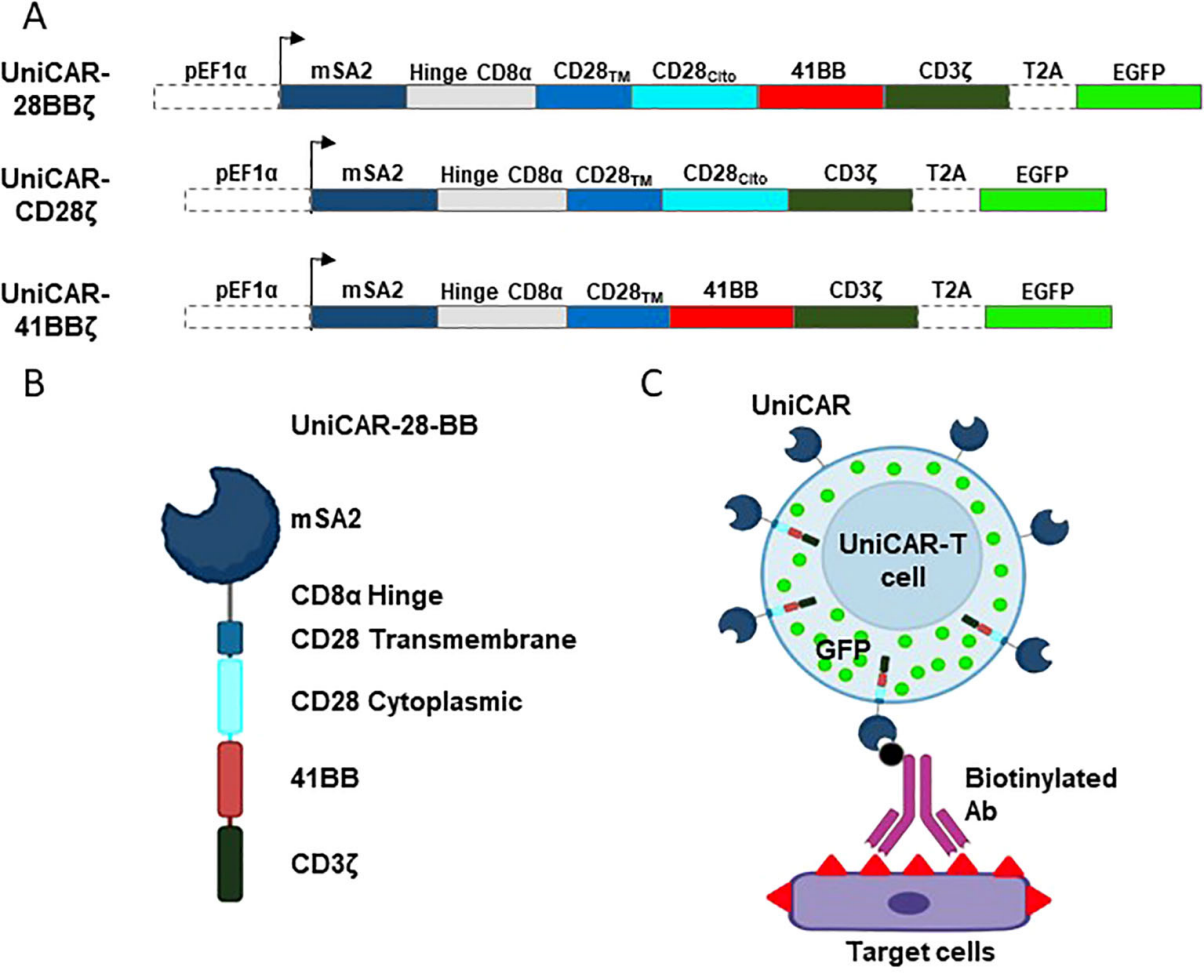
Schematic design of the vectors, UniCARs and their recognition system. **(A)** Design of the lentiviral expression construct encoding the 3rd generation UniCAR-28-BB, both 2nd generation UniCARCD28 and UniCAR41BB, and the eGFP. **(B)** Representation of the extracellular, transmembrane and intracellular elements that compose the 3rd generation UniCAR-28-BB. **(C)** Representation of a transduced cell expressing eGFP (green dots) and 3rd generation UniCAR-28-BB, which is able to recognize and bind to a biotinylated intermediate, such as a biotinylated monoclonal antibody. The biotinylated intermediate recognizes the antigens at the surface of the target cell. Figure created with BioRender.

Driven by the EF1a promoter, UniCAR extracellular recognition domain consists in the mSA2 sequence (17) followed by the CD8a-hinge spacer domain, the CD28 transmembrane domain, either CD28, 4-1BB or both cytoplasmic domains and, finally, the CD3ζ cytoplasmic domain (**Figure 1A**). eGFP gene was encoded in the same lecture frame, but it was separated from UniCAR by the T2A co-translation peptide sequence (**Figure 1A**). The structure of the resulting protein is shown in **Figure 1B**. Once the constructs were transduced into the desired cells, mSA2-UniCAR-T cells need a biotinylated intermediary, such as an Ab, to recognize the target cell (**Figure 1C**).

The third-generation pUniCAR-mSA2-CD28-41BB-CD3ζ-eGFP was provided by Creative Biolabs (Frankfurt, Germany), based on the published sequences (17). Second-generation pUniCAR-mSA2-CD28-CD3ζ-eGFP and pUniCAR-mSA2-41BB-CD3ζ-eGFP were synthetized by the deletion of 41BB and CD28 regions, respectively, from the third-generation pUniCAR-mSA2-41BB-CD28-CD3ζ construct. To accomplish these depletions, Q5^®^Site-Directed Mutagenesis Kit (New England Biolabs, NEB, Ipswich, MA, USA) was used, following manufacturer instructions and the corresponded primers (**Supplementary Figures S1A, B**).

The second-generation anti-CD19 CAR (CAR19) T cells, which consist of the A3B1 scFv, CD8 transmembrane domain, 4-1BB co-stimulatory domain, and CD3ζ stimulatory domain, was used as a positive control for CD19 target recognition and anti-tumor functionality of T cells (19, 21).

Lentiviral vectors were generated through the co-transfection of HEK-293T cells with various plasmids:pUniCAR-mSA2-41BB-CD28-CD3ζ, pUniCAR-mSA2-CD28-CD3ζ, pUniCAR-mSA3-41BB-CD3ζ, pCD19 (containing the full-length sequence of CD19) or pCAR-CD19, in combination with psPAX2 (RRID: Addgene_12260), pRSV-Rev (RRID: Addgene_12253), and pMD2.G (RRID: Addgene_12259, all plasmids were a gift from Prof. Dr. Trono, Addgene, Watertown, MA, USA). Transfection was performed using a calcium phosphate transfection kit (Sigma-Aldrich). The physical titers of the vectors were assessed after 0.45 µm filtration (Corning, Corning, NY, USA) by quantifying HIV-1-p24gag levels using an ELISA kit (Abcam, Cambridge, UK). The resulting lentivectors were named UniCAR-28-BB (3rd generation), UniCARCD28, UniCAR41BB, CD19, and CAR19, respectively. For lentivectors encoding eGFP, transduced cells were assessed by flow cytometry and sorted based on their eGFP expression.

### 2.4 UniCAR neutralization assay

To test de biotin accessibility of mSA2-UniCAR-28-BB, human Jurkat and Jurkat-UniCAR-28-BB cell lines were cultured in different culture media, such as RPMI 1640 medium or in X-vivo15 medium, both media supplemented with either 10% FBS + 1% antibiotic mix or with 5% of human ABS. The fluorochrome Atto-655 bound to a Biotin (Atto-655-Biotin, Sigma-Aldrich) was used to specifically label the extracellular mSA2 of the UniCAR expressed by Jurkat- UniCAR-28-BB cells.

### 2.5 *In vitro* activation and co-culture experiments

P96 well U-bottom plate was set-up with 20,000 non-engineered or engineered primary T cells cultured either alone or co-cultured with 20,000 target cells (K562, K562-CD19 or Raji cell lines) in complete RPMI 1640 medium and with 1.5 µg/ml of anti (α)-CD19 biotinylated Ab (Miltenyi Biotec Cat# 130-113-644, RRID: AB_2726197) or αCD20 biotinylated Ab (Miltenyi Biotec Cat# 130-111-336, RRID: AB_2656082). Non-biotinylated αCD19 Ab (Miltenyi Biotec Cat# 130-122-301, RRID: AB_2801882) and non-specific IgG biotinylated Ab (Miltenyi Biotec Cat# 130-119-877, RRID: AB_2751902) were used as control conditions, and the negative control condition was performed without any antibodies. Target cells were previously stained with Cell Trace Violet (CTvio) following the manufacturer’s recommendations (Invitrogen) to facilitate their visualization by flow cytometry.

After 72 hours of co-culture, cells were washed and stained with fluorochrome-bound antibodies against T cells and activation markers (CD3, CD4, CD8, CD25, CD71, ICOS and PD-1) to assess their expression by flow cytometry. Following surface labeling, effector engineered T cells were identified as CTvio-negative cells, and these activation markers were measured in CTvio-negative living cells, which were identified by the absence of staining with 7-Amino Actinomycin D (7-AAD, Invitrogen).

Additionally, to assess the cytotoxic activity performed by effector cells on target cells, co-cultured cells were directly stained with 7-AAD in the co-culture well. The viability of target CTvio-positive cells was measured by flow cytometry, with dead cells identified by positive 7-AAD staining. Cytotoxicity was calculated using the equation: 100 * (% of dead cells of experimental condition – % of dead cells of Cytotoxicity Negative control)/(% of dead cells of Cytotoxicity Positive control – % of dead cells of Cytotoxicity Negative control) (17). The Cytotoxicity Positive Control was K562, K562-CD19 or Raji cultured alone + 5% DMSO, and the Cytotoxicity Negative Control was K562, K562-CD19 or Raji cultured alone or with intermediary antibodies (depending on the culture condition).

### 2.6 Flow cytometry staining

As previously commented, cells were stained using anti-CD3 VioGreen^®^(Miltenyi Biotec Cat# 130-115-972, RRID: AB_2751292), anti-CD8 BV570 (BioLegend Cat# 100740 (also 100739), RRID: AB_2563055), anti-PD-1 PE (Miltenyi Biotec Cat# 130-120-382, RRID: AB_2752069), anti-CD4 ECD (Miltenyi Biotec Cat# 130-113-226, RRID: AB_2726037), anti-CD25 PE-Cy7 (Thermo Fisher Scientific Cat# 25-0259-42, RRID: AB_1257140), anti-ICOS BV650 (Biolegend Cat# 313550, RRID: AB_2749929) and anti-CD71 APC-Vio770 (Miltenyi Biotec Cat# 130-115-032, RRID: AB_2726862) Abs for 30 minutes at 4°C in staining buffer (PBS + 2% FBS). For the UniCAR labeling with Atto-655-Biotin, cells were incubated for 1 h at room temperature and in the dark in staining buffer. For the frequency of the CARCD19+ cells, we used FITC-Labeled Human CD19 (ACROBiosystems, Basel, Switzerland). Finally, 5-10 minutes before flow cytometry acquisition, cells were incubated with 7-AAD. Flow cytometry acquisition was done by MACSQuant Analyzer 16 - Flow Cytometer (Miltenyi Biotec) and the resulting data were analyzed using the Kaluza software (2.1 Version, Beckman Coulter, Brea, CA, USA, RRID: SCR_016182).

### 2.7 Statistics

Statistical analysis of the data was performed using GraphPad Prism 8 software (Dotmatics, Boston, MA, USA, RRID: SCR_002798) with the support of the Biostatistics Unit of the IiSGM. The measurements, their error deviations and the types of statistical methods used are specified in their respective figure captions. Statistical results with a p < 0.05 were considered statistically significant.

## 3 Results

### 3.1 Neutralization of UniCAR/biotin recognition by culture media

Due to the extracellular exposure of the mSA2, components of the culture medium could potentially block the biotin binding of intermediaries to UniCAR (22). Since the aim of the adoptive cell immunotherapy is to be used under physiological conditions, we tested how two commonly used culture media (RPMI 1640 and Xvivo15) supplemented with bovine or human serum affected the ability of the 3rd generation UniCAR-28-BB construct expressed on Jurkat cells to bind the biotin.

**Figure 2:**
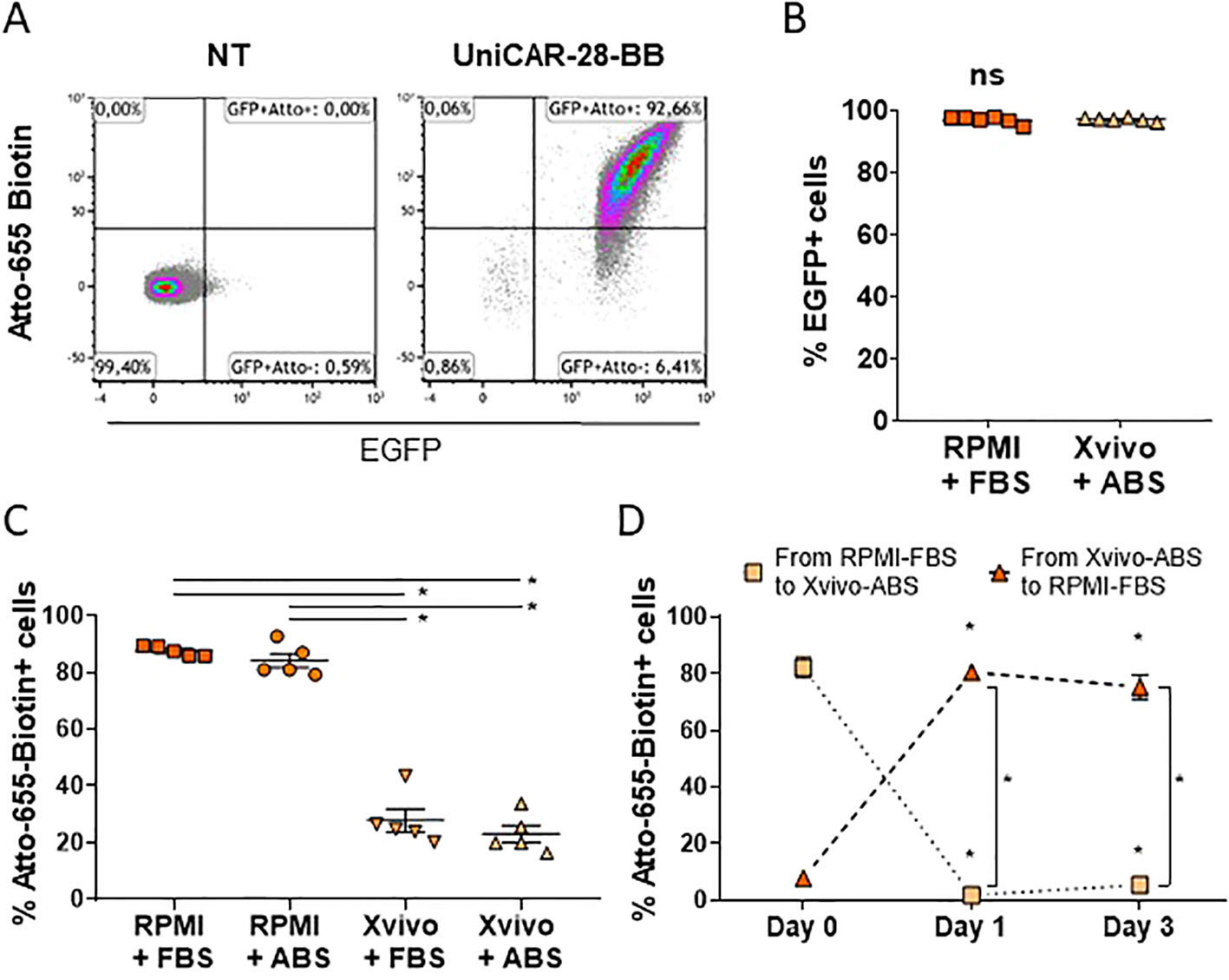
Components of culture media can neutralize the binding capacity of UniCAR to biotin. **(A)** Flow cytometry dot plots depicting non-transduced (NT) and transduced Jurkat cells with lentivectors encoding 3rd generation UniCAR-28-BB. Cells cultured in RPMI 1640 + 10% FBS were labeled with Atto-655 and followed by eGFP expression. These dot plots serve as a representative example from n=11. **(B)** Graph illustrating eGFP expression in UniCAR-28-BB Jurkat cells cultured in RPMI 1640 + 10% FBS and in Xvivo15 + 5% ABS, along with their mean + SEM. Ns= non-significant difference as determined by the Mann-Whitney comparison test. **(C)** Graph depicting the frequencies of Atto-655-Biotin-labeled UniCAR-28-BB-Jurkat cells when cultured in RPMI 1640 + 10% FBS or + 5% ABS and in Xvivo15 + 10% FBS or + 5% ABS, with their mean ± SEM. *Significant difference between conditions with p<0.05, as determined by the one-factor ANOVA multiple comparisons test. **(D)** Graph depicting the change in frequencies of Atto-655-Biotin-labeled UniCAR-28-BB Jurkat cells which had been cultured in RPMI 1640 + 10% FBS and moved to Xvivo15 + 10% FBS or had been cultured in Xvivo15 + 10% FBS and moved to RPMI 1640 + 10% FBS, with their mean ± SEM. *Significant difference between conditions with p<0.05, as determined by the 2way ANOVA multiple comparisons test.

Transduced Jurkat cells with the UniCAR-28-BB were sorted and detected by flow cytometry as eGFP+ cells. They also can be distinguished by the positive expression of Atto-655-Biotin binding to the mSA2 domain (**Figure 2A**). Sorted cells showed high eGFP expression, which was maintained independently of the culture media (**Figure 2B**). When cells were cultured in RPMI 1640, independently of its serum supplementation, the biotin union with the eGFP+ Jurkat-UniCAR-28-BB cells was maximum (**Figure 2C**). Notwithstanding, this ability to bind biotin was significantly compromised when cells were cultured in Xvivo15, independently of the serum source (**Figure 2C**). This result led us to infer that the Xvivo15 culture medium contains components unrelated to human serum, which hinder the binding.

Finally, to test if the biotin access to the UniCAR-28-BB could be restored, cells were moved from one medium to the other. While cells transferred from RPMI 1640 + FBS to Xvivo15 + ABS reduced their union ability to fluorescent biotin, cells previously cultured in Xvivo15 + ABS and subsequently cultured in RPMI 1640 + FBS significantly restored their Atto-655-biotin recognition capacity within 24h (**Figure 2D**). In summary, selecting the appropriate complete culture medium is crucial for studying streptavidin-based UniCAR cells. Based on these results, all subsequent experiments were performed using RPMI 1640 supplemented with 10% FBS. Additionally, the translation of UniCAR for potential human treatment should remain uncompromised, as the mSA2-biotin binding was not affected by the presence of human serum.

### 3.2 No alteration of the T cell phenotype after UniCARs transduction

**Figure 3:**
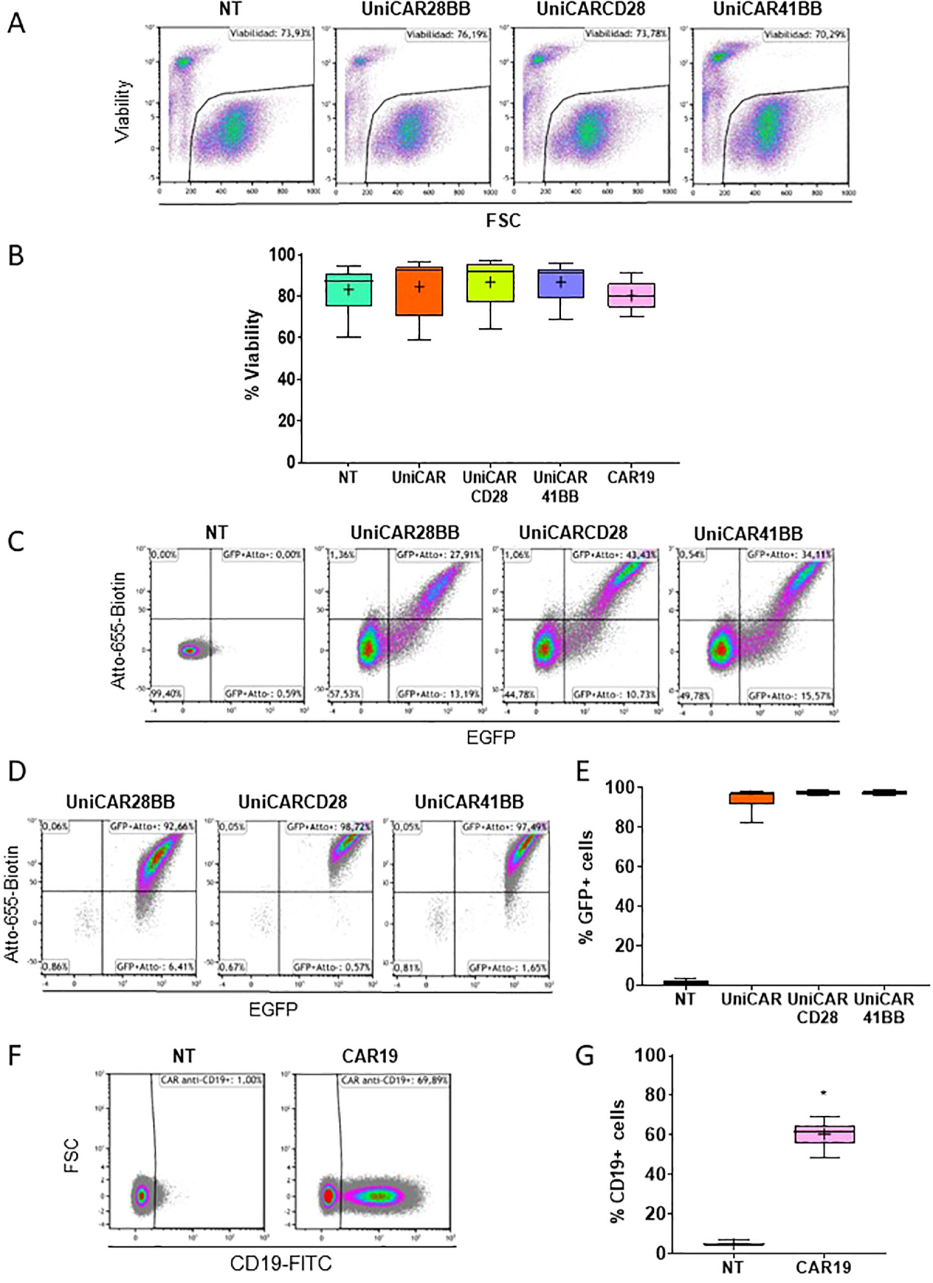
Viability and frequency of effector T cell transduction. **(A)** Flow cytometry dot plots representing the viability (visualized by the 7AAD negative signal) of Non-transduced (NT), UniCAR-28-BB, UniCARCD28, UniCAR41BB and CAR19 transduced T cells. These dot plots are a representative example of n=6. **(B)** Box and whiskers representing the frequency of viability of NT, UniCAR-28-BB, UniCARCD28, UniCAR41BB and CAR19 T cells, n=6. **(C)** Flow cytometry dot plots representing the frequencies of the eGFP and Atto-655 detection of NT, UniCAR-28-BB, UniCARCD28, and UniCAR41BB T cells before sorting. **(D)** Flow cytometry dot plots representing the frequencies of the eGFP and Atto-655 detection on NT, UniCAR-28-BB, UniCARCD28, and UniCAR41BB T cells after sorting. These dot plots are a representative example of n=6. **(E)** Box and whiskers representing the frequency of eGFP expression on NT, UniCAR-28-BB, UniCARCD28, and UniCAR41BB T cells, n=6. **(F)** Flow cytometry dot plots representing the frequency of CD19-FITC expression on NT and CAR19 T cells. These dot plots are a representative example of n=6. **(G)** Box and whiskers (10-90 percentile, the + symbol is the mean) representing the frequency expression of CD19-FITC on NT and CAR19 T cells, n=6.

After detecting the pivotal role of the culture medium, we transduced human PBMCs with UniCARs and CAR19 structures. These genetic modifications did not alter the cellular viability (**Figures 3A, B**). Following transduction, the expression of the three UniCARs structures was assessed via flow cytometry, with cells being identified as positive for UniCAR expression if they exhibited both eGFP+ and Atto-655-Biotin+ signals (**Figure 3C**). Subsequently, after sorting eGFP+ cells, the frequency of eGFP+ and UniCAR+ expressing cells exceeded 90% in every condition (**Figures 3D, E**). Since CAR19 construct did not have a selection marker, CAR19 transduced T cells could not be sorted without compromising its functionality. Nonetheless, 60.56 ± 2,89% (Mean ± SEM) of cells expressed CAR19 (**Figures 3F, G**).

As genetic modifications could potentially induce phenotypic changes in cells, we studied the phenotype of engineered T cells. In every UniCAR or CAR19 condition, the expression of the CD3 marker was found to be greater than 99% of living cells (**Supplementary Figure S2A**). Furthermore, we observed that genetic modification had no discernible effect on the frequency of CD4+, CD8+, double positive (DP, CD4+/CD8+), and double negative (DN, CD4-/CD8-) T cells (**Supplementary Figures S2B, C**). These frequencies remained consistent across all conditions, which is crucial as both CD4 and CD8 T cells contribute to anti-tumor activity (23).

### 3.3 Multi target anti-tumor efficacy of UniCARs

**Figure 4:**
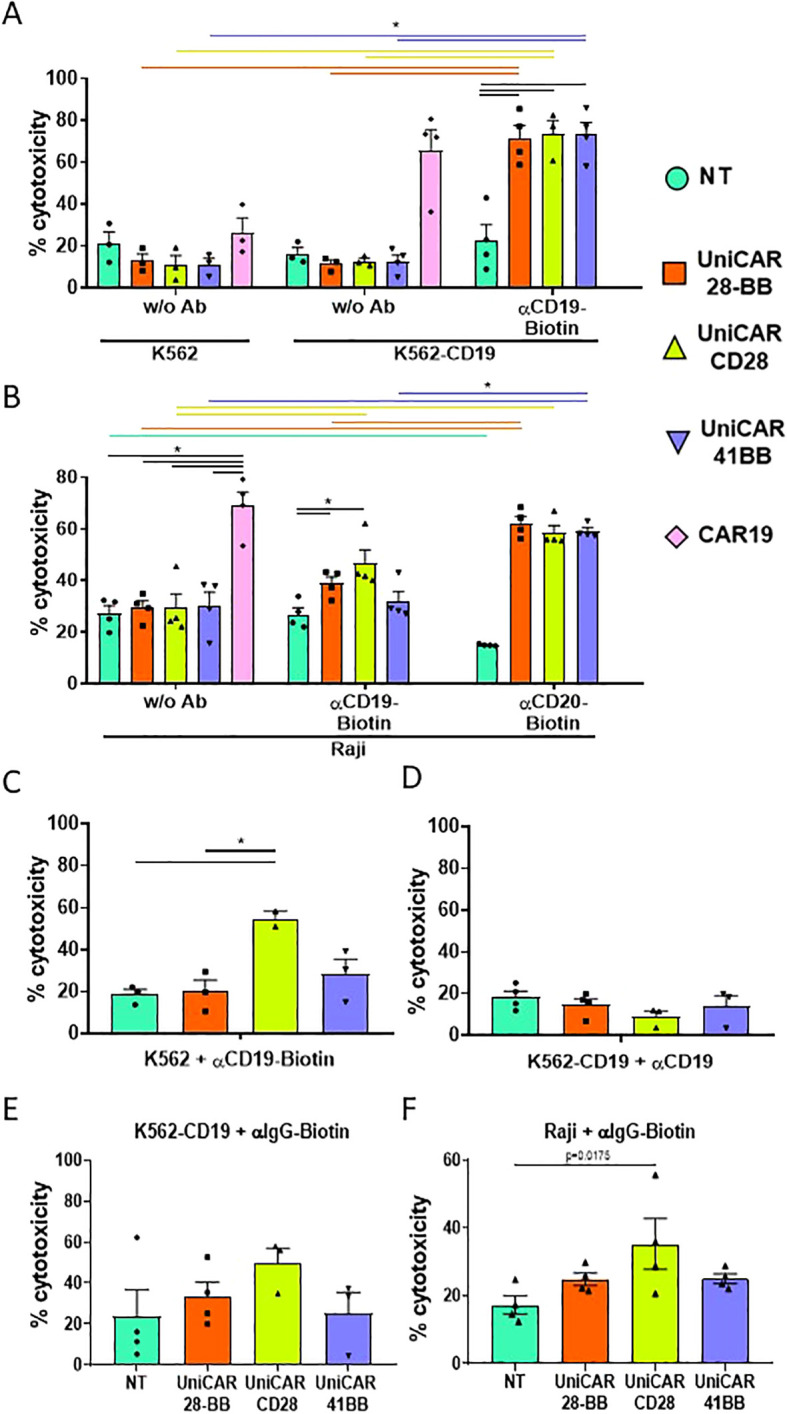
Cytotoxicity assay on target cell lines. **(A)** Histogram representing the frequencies of cell death (referred to as cytotoxicity, mean ± SEM) of CTVio+ K562 and K562-CD19 cells after 72 h of co-culture with Non-transduced T (NT), UniCAR-28-BB, UniCARCD28, UniCAR41BB and CAR19 T cells, both in the absence of intermediate antibody (w/o Ab) or with biotinylated αCD19 Ab. Significant differences were determined using a 2way ANOVA multiple comparisons, corrected with the Tukey’s test. **(B)** Histogram representing the cytotoxicity of CTVio+ Raji cells after 72 h of co-culture with NT, UniCAR-28-BB, UniCARCD28, UniCAR41BB and CAR19 T cells, in absence of intermediate antibody, with biotinylated αCD19 Ab, or with biotinylated αCD20 Ab (mean ± SEM). Significant differences were determined using a 2way ANOVA multiple comparisons, corrected with the Dunn’s test. **(C)** Histogram representing the frequencies of cell death (mean ± SEM) of CTVio+ K562 cells (which do not express CD19) after 72 h of co-culture with NT, UniCAR-28-BB, UniCARCD28, UniCAR41BB and CAR19 T cells, stimulated with biotinylated αCD19 Ab. Significant differences were determined using a 1way ANOVA multiple comparisons (Kruskal-Wallis’s test). **(D)** Histogram representing the frequencies of cell death (mean ± SEM) of CTVio+ K562-CD19 cells after 72 h of co-culture with NT, UniCAR-28-BB, UniCARCD28, UniCAR41BB and CAR19 T cells, stimulated with the non-biotinylated αCD19 Ab. Significant differences were determined using a 1way ANOVA multiple comparisons (Kruskal-Wallis’s test). **(E)** Histogram representing the frequencies of cell death (mean ± SEM) of CTVio+ K562-CD19 cells after 72 h of co-culture with NT, UniCAR-28-BB, UniCARCD28, UniCAR41BB and CAR19 T cells, stimulated with the non-specific biotinylated αIgG Ab. Significant differences were determined using a 1way ANOVA multiple comparisons (Kruskal-Wallis’s test). **(F)** Histogram representing the frequencies of cell death (mean ± SEM) of CTVio+ Raji cells after 72 h of co-culture with NT, UniCAR-28-BB, UniCARCD28, UniCAR41BB and CAR19 T cells, stimulated with the non-specific biotinylated αIgG Ab. Significant differences were determined using a 1way ANOVA multiple comparisons (Kruskal-Wallis’s test). All co-culture conditions were performed with a ratio 1:1 (effector T cell: Target cell). Each data point represents one experiment. *: Significant differences between indicated conditions are depicted by black lines, and significant differences inter-conditions are indicated by colored lines which correspond to the histogram’s color code. Inter-conditions statistics for **(C–F)** were depicted on **Supplementary Figure S4**. Significant differences when p<0.05.

Once the viability and phenotype of the engineered T cells were verified, the effector cell cytotoxicity was assessed by co-culturing engineered T cells with K562 and K562-CD19 cells, after evaluating the CD19 expression on modified K562-CD19 cells (**Supplementary Figure S3A**). As CAR19-T cells did not need any intermediary to exert their function, their elevated ability to kill is solely triggered by the direct recognition of the CD19 target protein on K562-CD19 cells, while maintaining low cytotoxicity against K562 negative for CD19, as expected (**Figure 4A**). There were also no differences in the low cytotoxic capacities of any of the UniCAR-T cells when they were co-cultured with K562 and K562-CD19 in the absence of any intermediary. However, when all UniCAR-T cells were co-cultured with K562-CD19 and αCD19 biotinylated Ab, their cytotoxicity was significantly increased, being comparable to CAR19-T cells (**Figure 4A**).

To test the multi target capacity of the UniCAR, we co-cultured engineered T cells with Raji cell line, which equally expressed CD19 and CD20 markers (**Supplementary Figures S3A, B**). CAR19-T cells showed the same cytotoxic ability on Raji as on K562-CD19, as expected (**Figure 4B**). When αCD19 biotinylated Ab was added, UniCAR-28-BB and UniCARCD28-T cells exhibited a slight, but significant, increase in their cytotoxicity towards Raji cells compared to non-transduced (NT) T cells. On the other hand, UniCAR41BB-T cell condition did not show any clear cytotoxicity increase. Furthermore, each UniCAR-T subset demonstrated significantly greater cytotoxic capacities when utilizing αCD20 biotinylated antibody compared to NT-T cells when co-cultured with Raji cells. The cytotoxicity observed in Raji + anti-CD20-Biotin + UniCAR-T cells reached the level of cytotoxicity observed in Raji + CAR19-T cells (**Figure 4B**). To validate the specificity of UniCAR-T cell cytotoxicity dependent on the UniCAR and the correct biotinylated intermediary, we co-cultured engineered T cells with K562 cells lacking CD19 expression, supplemented with αCD19 biotinylated antibody. Results showed that although UniCAR41BB-T cells induced a slight increase in cytotoxicity, it was not significant. However, UniCARCD28-T cells induced toxicity in K562 cells despite their lack of CD19 on their surface, upon addition of the antibody (**Figure 4C**, **Supplementary Figure S4** for the statistical representation of inter-condition differences). Conversely, none of the UniCAR-T cell subsets induced death in K562-CD19 cells when co-cultured with non-biotinylated αCD19 antibody (**Figure 4D**). Thus, these findings suggest that the presence of biotin alone was sufficient to activate the cytotoxic activity of UniCARCD28-T cells, independently of specific target antigen engagement. To further explore this non-specificity phenomenon, UniCAR-T cells were co-cultured with K562-CD19 or Raji cells in the presence of a non-specific biotinylated IgG, which does not recognize either CD19 or CD20 markers. The presence of non-specific biotinylated IgG increased significantly the UniCARCD28-T cell cytotoxic activity compared with the NT condition in Raji cell cocultures, but not with K562-CD19 cells (**Figures 4E, F**, **Supplementary Figure S4** for the statistical representation of inter-condition differences). The co-culture of K562-CD19 or Raji cells with non-specific biotinylated IgG did not significantly increase the cytotoxicity of UniCAR-28-BB-T and UniCAR41BB-T cells (**Supplementary Figure S4**).

In summary, UniCAR-T cells showed efficacy in inducing cell death in two different target cell lines, achieving cytotoxicity levels nearly comparable to classical CAR19-T cells, depending on the target antigen. However, second-generation UniCARCD28-T cells displayed non-specific cytotoxicity when exposed to any biotinylated antibodies, regardless of the presence of the target marker on the cell surface.

### 3.4 Third-generation UniCAR-28-BB-T cell activation was more specific than both second-generation UniCAR-T cells

**Figure 5:**
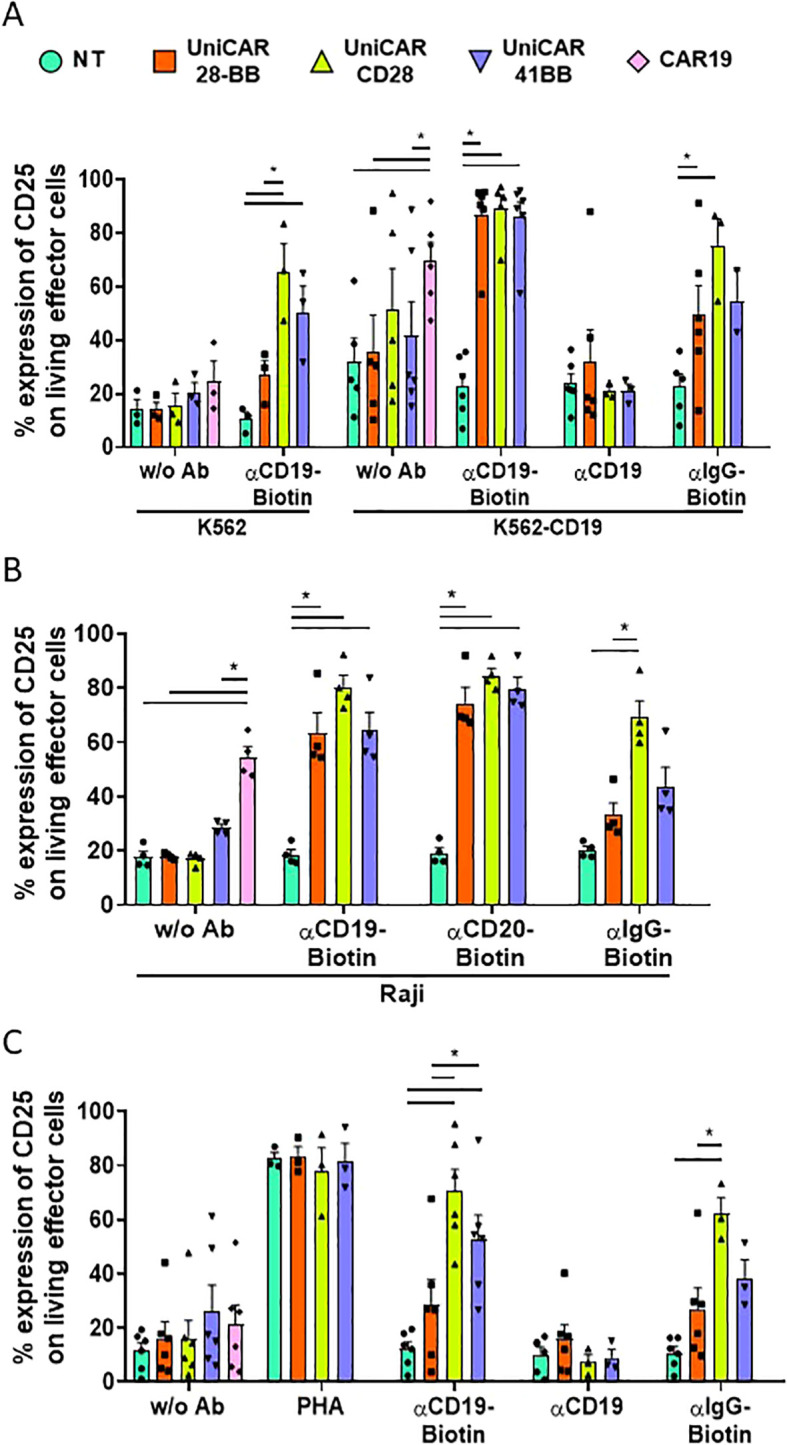
Activation of effector T cell subsets measured by CD25 expression. **(A)** Histogram representing the frequencies of CD25+ cells on NT, UniCAR-28-BB, UniCARCD28, UniCAR41BB and CAR19 T cells (gated on living cells, mean ± SEM) after 72 h of co-culture with CTVio+ K562 and CTVio+ K562-CD19 cells, in the absence of antibody intermediate, or with biotinylated αCD19 Ab, non-biotinylated αCD19 Ab or with biotinylated IgG. *Significant differences between indicated conditions. Inter-conditions statistics for **(A)** were depicted on **Supplementary Figure S6A**. **(B)** Histogram representing the frequencies of CD25+ cells on NT, UniCAR-28-BB, UniCARCD28, UniCAR41BB and CAR19 T cells (gated on living cells, mean ± SEM) after 72 h of co-culture with CTVio+ Raji cells, in the absence of intermediate antibody, or with biotinylated αCD19 Ab, biotinylated αCD20 Ab or with biotinylated IgG. *Significant differences between indicated conditions. Inter-conditions statistics for **(B)** were depicted on **Supplementary Figure S6B**. **(C)** Histogram representing the frequencies of CD25+ cells on NT, UniCAR-28-BB, UniCARCD28, UniCAR41BB and CAR19 T cells (gated on living cells, mean ± SEM) after 72 h of culture alone in the absence of intermediate antibody, or with PHA, biotinylated αCD19 Ab, biotinylated αCD20 Ab or biotinylated IgG. *Significant differences between indicated conditions. Inter-conditions statistics for **(C)** were depicted on **Supplementary Figure S6C**. Significant differences when p<0.05, as determined by the by 2way ANOVA multiple comparisons test, corrected with the Tukey’s test. All co-culture conditions were performed with a ratio 1:1 (effector T cell: Target cell). Each data point represents one experiment.

CAR-T cell activity is related to the ability to recognize their specific targets and subsequently to be activated through CAR intracellular signaling domains (24). Given that UniCARCD28-T cells exhibited non-specific cytolytic activity, understanding their activation patterns becomes crucial. We first observed the expression of the CD25 marker on effector UniCAR-T cells when co-cultured with K562 or K562-CD19 target cell lines (**Supplementary Figure S5**). As it was expected, the frequency of CD25+ in conventional CAR19-T cells increased when they were in contact with K562-CD19 (69.8 ± 6.5%, % ± SEM) but not with K562 (24.6 ± 7.4%, % ± SEM, **Figure 5A**, **Supplementary Figure S5**, and **Supplementary Figure S6A** for the statistical representation of inter-condition differences). UniCAR-28-BB-T cells exhibited a basal activation level, as evidenced by CD25 expression without Ab (in K562: 14.1 ± 2.7%, in K562-CD19: 35.5 ± 13.8%, % ± SEM), similar to NT-T cell co-culture conditions with K562 (14.1 ± 3.7%), or K562-CD19 (31.8 ± 12.0%, % ± SEM). However, when biotinylated αCD19 Ab was added in the culture medium with the specific target K562-CD19 cells, frequency of CD25+ UniCAR-28-BB-T was significantly higher than the CD25 level in the NT control condition (86.8 ± 6.0% and 22.8 ± 4.6%, % ± SEM, respectively, **Figure 5A**).

Although the frequencies of CD25 expression levels on UniCAR41BB (85.8 ± 5.8%) and UniCARCD28-T cells (89.1 ± 4.9%) were similar to UniCAR-28-BB-T cells under the experimental condition of K562-CD19 + biotinylated-αCD19 antibody, they also exhibited higher CD25 expression in several control conditions, especially UniCARCD28-T cells. Thus, UniCARCD28-T cells showed a nonspecific CD25 expression in the co-culture with K562 + biotinylated αCD19 Ab (65.6 ± 10.4%), K562-CD19 without Ab (51.1 ± 15.5) and K562-CD19 + biotinylated IgG (75.0 ± 10.3%, % ± SEM), while CD25 expression of UniCAR41BB-T cells was only elevated in K562 + biotinylated αCD19 Ab condition (50.3 ± 9.8%, % ± SEM, **Figure 5A**). The expression level of CD25 was also measured on CD25+ living effector cells using Mean Fluorescence Intensity (MFI, see **Supplementary Figure S7A**). Although there were non-specific increases in the frequency of CD25+ cells in some culture control conditions (**Figure 5A**), only the K562-CD19 + αCD19-Biotin condition induced a significant increase in CD25 MFI on effector cells.

Similar results were also observed when UniCAR and CAR19-T cells were co-cultured with the Raji cell line. It is notable that the frequency of CD25+ cells bearing every UniCAR construct was increased, not only with biotinylated αCD19 antibody but also with biotinylated αCD20 antibody, confirming their multi-target recognition ability (**Figure 5B**, **Supplementary Figure S6B** for the statistical representation of inter-condition differences). In summary, 2nd generation UniCARCD28 and, to a lesser extent, UniCAR41BB-T cells exhibited nonspecific activity in the presence of their biotinylated ligand, regardless of whether the target marker was present on the objective cells (**Figure 5B**).

In patients, ideally, UniCAR-T cells should not be activated once the tumoral target cells have been eliminated, as sustained cellular activation could lead to hyper-activation of the immune system and exhaustion of the therapeutic cells. Therefore, we also studied the frequency of CD25+ effector T cells when cultured alone (with or without antibody intermediaries) to test if this phenomenon was also observable in the absence of target cells. It was seen that CD25+ frequency expression on UniCAR-28-BB-T cells remained at low levels, regardless of the antibody with which they were cultured or in the absence of antibody (with biotinylated αCD19 Ab; 28.5 ± 9.2%, with αCD19 non biotinylated; 15.8 ± 5.4%, with biotinylated αIgG; 26.8 ± 7.9%, and without Ab; 16.5 ± 6.0%, % ± SEM), as the NT negative control (with biotinylated αCD19 Ab; 12.1 ± 2.7%, with αCD19 non biotinylated; 10.2 ± 2.6%, with biotinylated αIgG; 10.7 ± 2.3% and without Ab; 11.5 ± 2.9%, % ± SEM, **Figure 5C**, **Supplementary Figure S6C** for the statistical representation of inter-condition differences). Therefore, in the absence of target cells, even in the presence of the specific biotinylated antibodies, UniCAR-28-BB-T cells did not exhibit significant activation. On the other hand, frequencies of CD25+ UniCAR41BB-T cells and, especially, CD25+ UniCARCD28-T cells exhibited significantly increases when cultured with biotinylated αCD19 antibody (52.8 ± 8.8% and 70.5 ± 8.0%, % ± SEM, respectively) or non-specific biotinylated IgG (38.3 ± 6.8% and 62.1 ± 6.0%, % ± SEM, respectively), even in the absence of any target cell, compared to NT and UniCAR-28-BB-T cells (**Figure 5C**).

**Figure 6:**
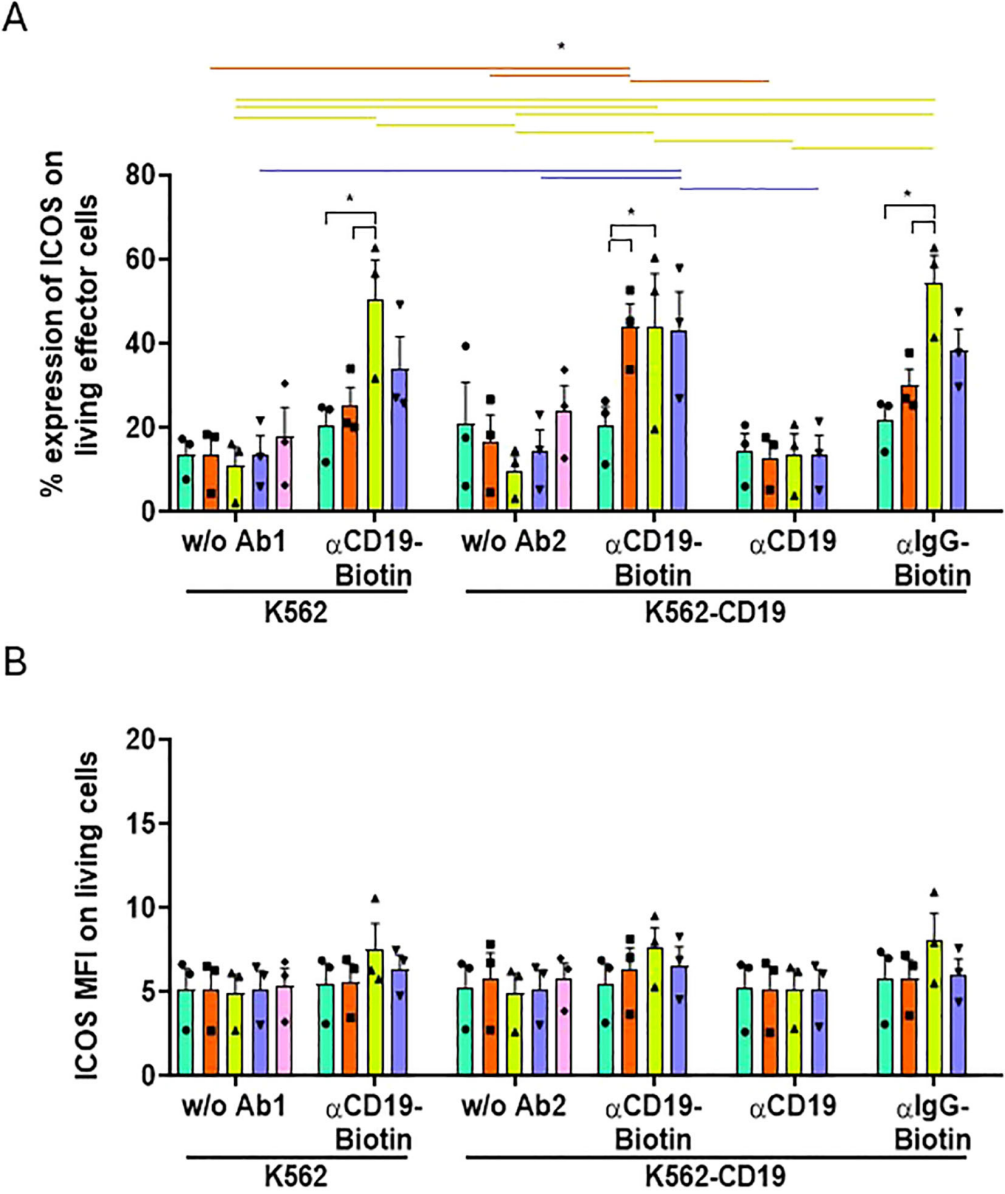
Activation of effector T cell subsets measured by ICOS expression. **(A)** Histogram representing the frequencies of ICOS+ cells on NT, UniCAR-28-BB, UniCARCD28, UniCAR41BB and CAR19 T cells (gated on living cells, mean ± SEM) after 72 h of co-culture with CTVio+ K562 and CTVio+ K562-CD19 cells, in the absence of antibody intermediate, or with biotinylated αCD19 Ab, non-biotinylated αCD19 Ab or with biotinylated IgG. *: Significant differences between indicated conditions are depicted by black brackets, and significant differences inter-conditions are indicated by colored lines which correspond to the histogram’s color code. **(B)** Histogram representing the Mean of Fluorescent Intensity (MFI) of ICOS+ cells on NT, UniCAR-28-BB, UniCARCD28, UniCAR41BB and CAR19 T cells (gated on living cells, mean ± SEM) after 72 h of co-culture with CTVio+ K562 and CTVio+ K562-CD19 cells, in the absence of antibody intermediate, or with biotinylated αCD19 Ab, non-biotinylated αCD19 Ab or with biotinylated IgG. Significant differences when p<0.05, as determined by the by 2way ANOVA multiple comparisons test, corrected with the Tukey’s test. All co-culture conditions were performed with a ratio 1:1 (effector T cell: Target cell). Each data point represents one experiment.

All these results were confirmed by observing a similar expression pattern for other activation markers, such as ICOS (**Figure 6**). ICOS is an activation marker and has already been identified as a potential indicator of CAR-T immunotherapy success (25). The frequency of cells expressing ICOS, like CD25, increased when all UniCARs T-cells were co-cultured with K562-CD19 + biotinylated-αCD19 (**Figure 6A**), although their MFI did not change (**Figure 6B**). Notably, the frequency of ICOS+ UniCARCD28-T cells exhibited significant non-specific increases when cultured with K562 and biotinylated-αCD19 (50.34 ± 9.5%, % ± SEM), or with K562-CD19 and non-specific biotinylated-IgG (54.35 ± 6.5%), compared to the NT condition with the biotinylated-IgG (21.70 ± 3.7%, % ± SEM, **Figure 6A**). Other activation markers, CD71 and PD-1, were analyzed and showed a similar pattern to CD25 on UniCAR-T cells and NT-T cells when exposed to K562, K562-CD19 or Raji cells (**Supplementary Figure S8**) or when cultured alone without target cells (**Supplementary Figure S9**). However, their MFI was significantly increased only with the specific combination of K562-CD19 + biotinylated-αCD19 (**Supplementary Figures S7B, C**).

**Figure 7:**
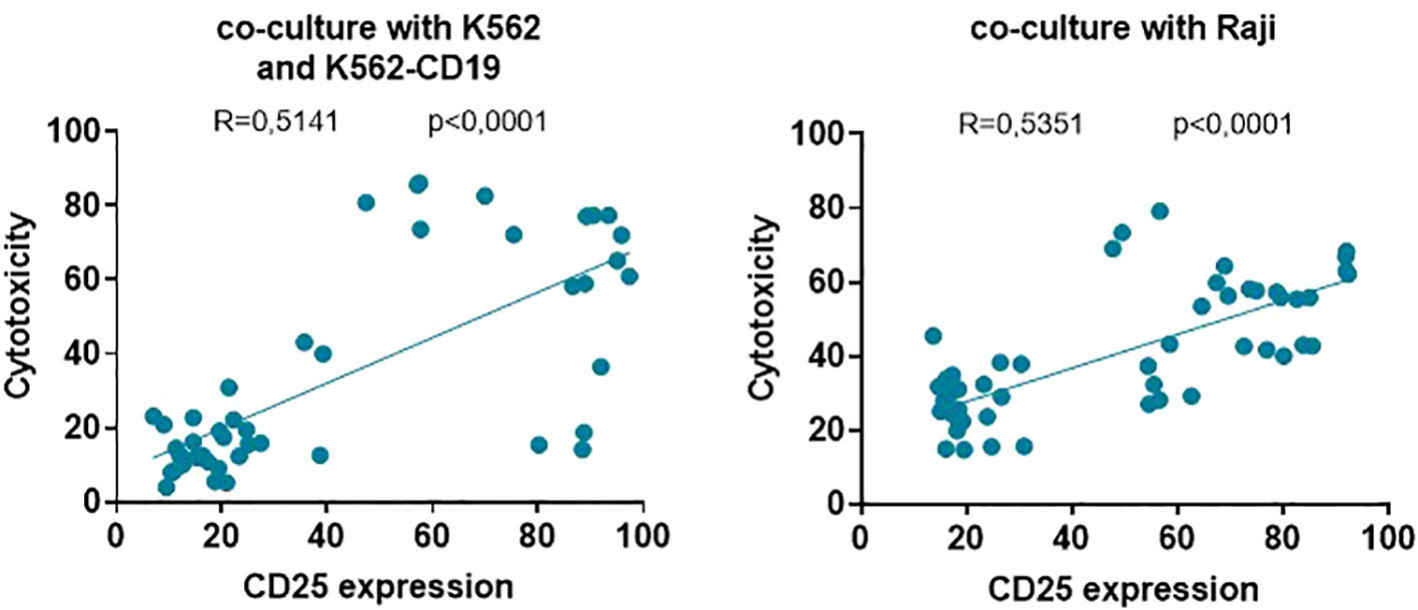
Correlations between effector T cell activation and cytotoxicity of target cell lines. Correlations and linear regression between the frequency of effector T cells positive for the CD25 marker and the percentage of cytotoxicity of K562 and K562-CD19 cells (left) and Raji cells (right). Correlations were determined by Pearson’s rank correlation and considered statistically significant when p < 0.05. Each symbol corresponds to one culture condition.

Finally, the significant correlation and linear regressions between effector T cell activation (CD25 expression) and their cytolytic activity over K562, K562-CD19 (r=0.5141; p<0.0001) and Raji (r=0.5351, p<0.0001) target cells showed that the higher the activation induced by each UniCAR or CAR19 on T cells, the higher the cytotoxic ability against both cell lines used in this study (**Figure 7**). Therefore, the specific activation of UniCAR-28-BB-T cells and the non-specific activation of UniCARCD28- and UniCAR41BB-T cells could preclude their cytotoxic capacity.

## 4 Discussion

The use of mSA2-UniCAR structures may overcome some limitations observed in classical antigen-specific CAR structures, such as modulation of functionality based on the presence or absence of an intermediate. However, incorporating a third element, such as biotinylated intermediates, introduces new potential impediments. Indeed, as we have observed, culture conditions could impair the availability of biotin recognition. While the classical complete medium culture RPMI 1640 supplemented with human serum did not inhibit the mSA2/Biotin interaction, another commonly used culture medium containing components not defined by the manufacturer abolished this union. The presence of biotin in culture media might affect the binding of UniCAR-cells to their biotinylated intermediates. However, according to the manufacturer’s information, the biotin concentration in the RPMI 1640 medium used in this study is 200 ng/mL, whereas circulating serum concentrations of biotin in the general population typically range from 0.1 ng/mL to 0.8 ng/mL (26). Therefore, it is unlikely that this biotin concentration would hinder the proper mSA2-UniCAR/biotin binding. Therefore, as this phenomenon is independent of serum supplementation, we do not foresee any difficulties in future animal models or in its translation to human applications.

Other essential factor to consider is that, for successful therapeutic approaches using CARs, the appropriate co-stimulatory domain, or combination thereof, must be selected for each strategy (10). In fact, understanding how the structure of a UniCAR affects the T cell function is critical to guide their clinical implementation. In this study, second generation UniCARCD28- and UniCAR41BB-T cells presented a nonspecific activity when in contact with a biotinylated intermediate, regardless of their antigen specificity or the presence of target cells, while third generation UniCAR-28-BB-T cells were only activated by the correct combination of the target cell and the specific biotinylated intermediary.

The difference in the combination of the UniCAR’s co-stimulation domains likely explains the differences in recognition specificity, which in turn influence the cellular activation pattern and cytotoxic ability. In T cells, conventional CARs based on CD28 are characterized by intense cellular activation, consequently leading to rapid cytolytic capacity and tumor elimination, both of which are significantly higher than those exerted by CAR41BB (27–31). Conversely, CAR structures based on 4-1BB mediate a more sustained cellular activation, enhancing proliferation and mitigating exhaustion of effector cells compared to CAR-T cells based on CD28 (9, 28, 31–33). In non-conventional CAR-T approach such as UniCAR-T cells, which need intermediaries to be activated, the correlation between specificity for the intermediaries and the cellular functionality must be extensively studied to avoid non desired cellular hyper-activation. Sun et al. described that the origin of the differences between these two co-stimulatory signals could be attributed to the kinase LCK, recruited by the joint action of the CAR and the co-receptors CD4 or CD8. This group reported that the phosphorylation sites that this LCK kinase has on the CD3-ζ chain are basally phosphorylated in CAR constructs with the CD28 sequence, triggering a highly elevated and permanent tonic activation signal promoted by an increase in the glycolysis metabolic pathway (34).

We hypothesized that the tonic basal activation of CARCD28-T cells could contribute to their non-specificity. Indeed, transmembrane and intracellular CD28 CAR domains are known to induce a heterodimerization with endogenous CD28 and increase homodimers of endogenous CD28, which may induce off-target activation (35). Additionally, the formation of CAR clusters can lead to antigen-independent tonic signaling and subsequent cellular activation (9, 36). In our study, we observed by flow cytometry that median fluorescence intensity of UniCARCD28 and UniCAR41BB structures expression (subset eGFP+ Atto-655-Biotin+) was higher than that of UniCAR-28-BB expression in T cells. The increased presence of UniCAR proteins on the cell surface may promote the formation of such UniCAR clusters, potentially leading to nonspecific subsequent activation (**Figures 3C, D**). This hypothesis is supported by a comparative study of CD28, 4-1BB or CD28 + 4-1BB based CAR-T cells that showed that the CAR-CD28 structures generate large clusters with high CAR dimers density at the surface of the cells and hence participate in the elevated cellular activation, possibly lowering the activation threshold (9, 37). Moreover, the UniCARCD28 clusters may increase UniCAR sensitivity for low abundant antigens or, in our case, for biotin regardless of the target cells presence (37, 38). Therefore, this enhanced tonic activation state could explain why the intense cellular activation cascade is induced after contact only with the biotinylated antibody in UniCARCD28-T cells, while UniCAR41BB-T cells showed lower nonspecific activation. Since there was correlation between the cellular activation and the cytotoxic capacity, the tonic stimulation of UniCARCD28-T cells is likely associated with their non-specific ability to kill co-cultured cells. On the other hand, we cannot exclude the possibility that the lower MFI of UniCAR-28-BB on the surface of effector cells, likely due to the size of the construct affecting its transduction ability, could explain the lack of non-specific activity observed with this construct compared to the second-generation UniCARs. The lower presence of UniCAR-28-BB may decrease its sensitivity to low-abundance antigens, such as biotin in our case, and consequently increase the minimum threshold required for activation signals. Tonic activation could potentially lead to cytokine release syndrome or immune cell-associated neurotoxicity syndrome in patients undergoing adaptive CAR-T therapy. These results are partially contradictory with those shown by Lohmueller et al. (17). Although they demonstrated that their mSA2-UniCAR-T cells with CD28 or 4-1BB increased their expression of CD69 when cultured with specific biotinylated Ab, they did not observe activation of UniCARCD28 or UniCAR41BB-T cells in the presence of a biotinylated antibody for a non-relevant target. This difference with our second generation UniCAR-T cells cannot be explained by the structure of the UniCAR since they have the same CAR sequences under the same promoter. On the other hand, the backbone vectors differ, and it is known that vector structures can influence the transduction efficiency. Our vectors may allow for an increased number of integrated molecules, thereby increasing the number of UniCARs on the cell surface. As previously mentioned, the quantity of these molecules might affect the response to the target antigen by either lowering or raising the activation threshold. Without comparing the vector structures directly, we cannot fully explain the observed differences in specificity for activation. However, our findings may be pivotal and could justify a more in-depth analysis.

In our efforts to combine the potent cytotoxicity of CD28-based CARs with the reduced exhaustion and long-term persistence of 4-1BB-based CARs, we observed that third generation UniCAR-28-BB-T cells exhibited significant activation only in the presence of the correct biotinylated intermediary antibody and the appropriate target cells. However, in this study, the exhaustion of effector cells was not examined and should be addressed in future research, given its significance in CAR-T immunotherapy approaches.

Furthermore, this specific cytolytic capacity was observed with two different tumor cell lines, K562-CD19 and Raji, targeting two different antigens, CD19 and CD20. The activation of UniCAR-T cells, as assessed by measuring CD25, ICOS, CD71, and PD-1 using flow cytometry, was specific to co-culturing with Raji cells and the specific biotinylated antibodies. However, it is important to note that the cytotoxic activity of all UniCAR-T cells combined with biotinylated-αCD19 was lower against Raji cells compared to that observed with biotinylated-αCD20. This discrepancy may be explained by the downregulation of CD19 that might impede subsequent cell death. To support this hypothesis, previous studies have shown that in co-culture experiments with Raji cells and classical αCD19 or αCD20 CAR-T cells, there is a rapid down-modulation of full-length CD19 expression, and to a lesser extent, CD20 (39). Furthermore, when anti-CD19 or anti-CD20 antibodies were associated with liposomes to develop an intracellular drug delivery system targeting B cells, anti-CD19 was more effective, as it was internalized more rapidly than anti-CD20, which was considered to be a non-internalizing antibody (40). The rapid and extensive down-modulation of CD19 expression at the cell surface could impair target cell recognition by effector cells, allowing Raji cells to evade cell death and continue replicating. However, the cytotoxic capacity of UniCAR-28-BB-T cells did not surpass that exerted by both second generation UniCARs-T cells, as reported by some authors in their animal model studies (41–44) or even in clinical trials (45). These studies suggested that the accumulation of multiple additional co-stimulatory signals might not induce enhanced cytotoxic capacity and could even lead to a detriment in cellular functionality. However, those studies primarily utilized conventional CAR structures, and the requirement for a biotinylated intermediary could potentially introduce differential flexibility to the entire UniCAR structure. It has already been demonstrated that each element of the CAR structure, not limited to their intracellular domains, can influence CAR signaling, thereby affecting activation and functionality. Hence, a comprehensive analysis of the individual components of the structure could aid in the design of future CAR structures that are both highly specific and functional.

Due to their therapeutic potential, two clinical trials (NCT03190278 and NCT04633148) based on a switchable universal CAR-T platform, where UniCAR-T cell activity depends on the presence of a soluble adapter, will start soon (46). These clinical trials demonstrate the significant interest in a flexible CAR structure for future immunotherapies. Our results suggest that UniCAR-28-BB-T cells could represent a highly specific therapeutic approach with potent anti-tumor capabilities, particularly highlighting their ability to target multiple antigens.

## Data Availability

The original contributions presented in the study are included in the article/**Supplementary Material**. Further inquiries can be directed to the corresponding author.
