# Supplementary material for: High specificity of engineered T cells with third generation CAR (CD28-4-1BB-CD3-ζ) based on biotin-bound monomeric streptavidin for potential tumor immunotherapy
Source: Front Immunol. 2024 Sep 19;15:1448752. doi: 10.3389/fimmu.2024.1448752 (PMC11446752; doi:10.3389/fimmu.2024.1448752)
Supplement: Supplementary file 1 [file DataSheet1.pdf]

# *Supplementary Material*

## High specificity of engineered T cells with third generation CAR (CD28-4-1BB-CD3-ζ) based on biotin-bound monomeric streptavidin for potential tumor immunotherapy

Jorge Gallego-Valle 1, Verónica Astrid Perez-Fernandez 1, Jesus Rosales-Magallares 1, Sergio Gil-Manso1, Maria Castellá 2, Europa Azucena Gonzalez-Navarro 2, Rafael Correa-Rocha 3, Manel Juan 2, Marjorie Pion *1.

*****Correspondence: marjorie.pion@iisgm.com

A

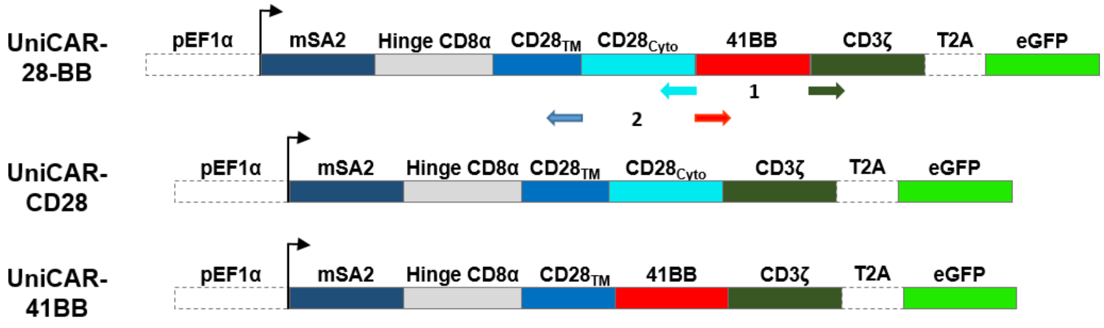


B

| 1 _ Primers to generate UniCAR28 (5´-3´) |
| --- |
| CGGGTGAAGTTCAGCAGAAGCG |
| CCCTCCGCCGGACCGGTAG |
| |
| 2 _ Primers to generate UniCAR41BB (5´-3´) |
| AAACGGGGCAGAAAGAAAC |
| CACCCAAAAGATGATGAAGG |

**Supplemental Figure 1: Schematic design of the UniCAR vectors and UniCARs structure**. (A) Schemas of the lentiviral constructs encoding the 3rd generation UniCAR-28-BB, 2nd generation UniCARCD28 and UniCAR41BB, and eGFP. Arrow: visualization of primers recognition site flanking the regions to be eliminated of the 3rd generation UniCAR-28-BB to create both 2nd generation UniCARCD28 and UniCAR41BB. (B) Primers sequences used to create 2nd generation UniCARCD28 and UniCAR41BB from the 3rd generation UniCAR-28-BB.

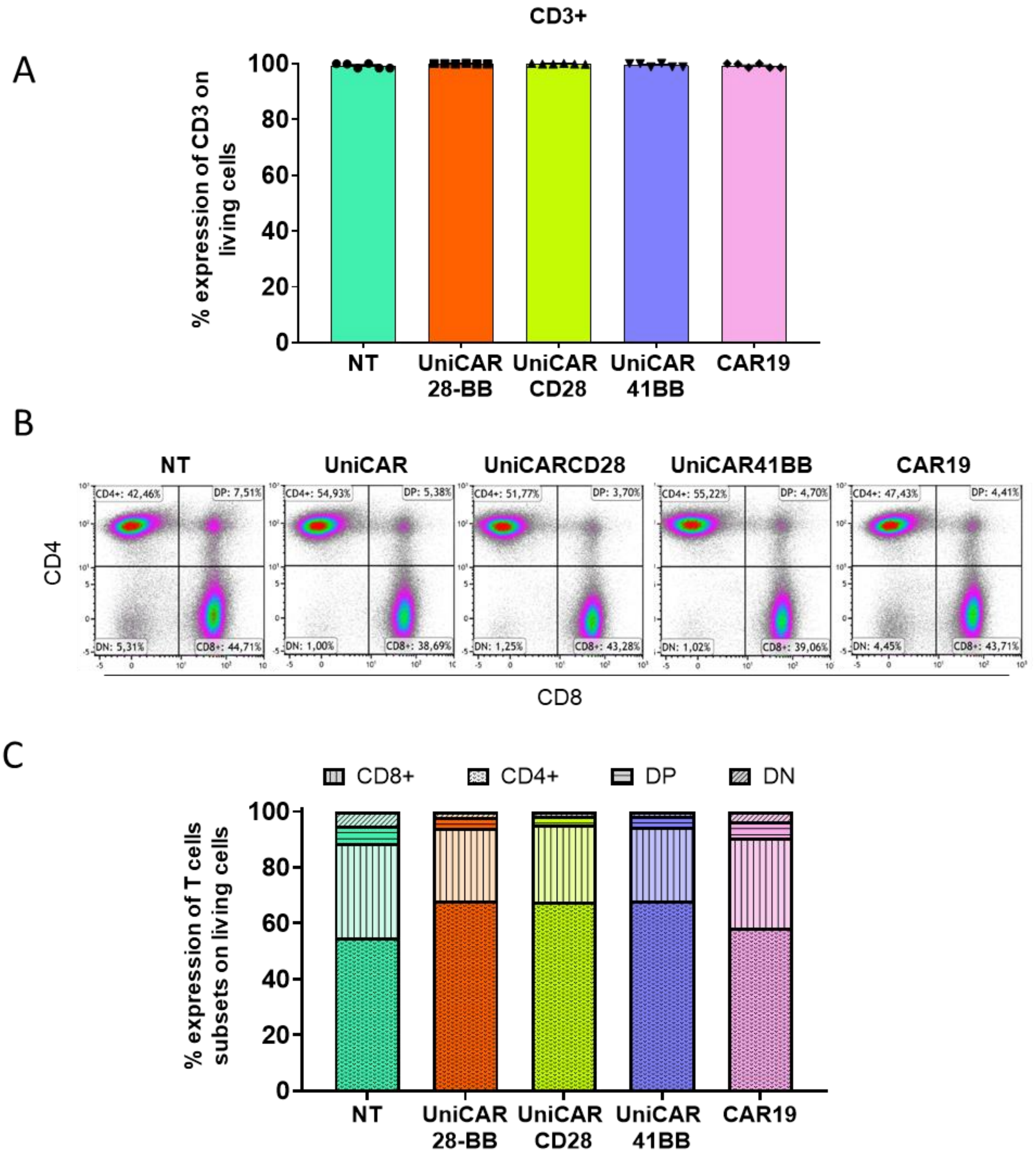


**Supplemental Figure 2: Phenotype of engineered effector T cells**. (A) Histogram representing the frequencies of CD3+ cells on non-transduced (NT), UniCAR-28-BB, UniCARCD28, UniCAR41BB and CAR19 T cells (gated on living cells, mean ± SEM) before setting up the co-culture experiments. Each data point represents one experiment. (B) Flow cytometry dot plots representing the CD4 and CD8 expression in NT, UniCAR-28-BB, UniCARCD28, UniCAR41BB, and CAR19 T cells. Dot plots representative example from a total of n=6. (C) Histogram representing the frequency of four subsets derived from CD4 and CD8 expression [CD8+, CD4+, CD4+CD8+ Double positive (DP) and CD4negCD8neg Double Negative (DN)] on NT, UniCAR-28-BB, UniCARCD28, UniCAR41BB and

CAR19 T cells (gated on living cells).

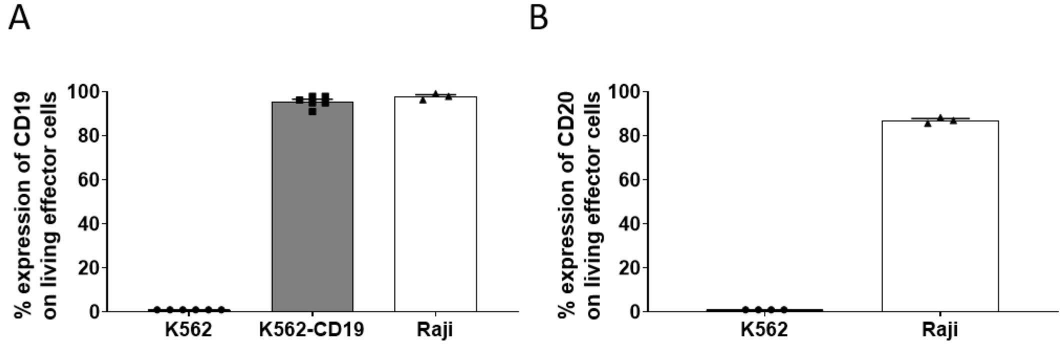


**Supplemental Figure 3: CD19 and CD20 expression of cell lines.** (A) Histogram representing the frequency of CD19 positive cells (gating on living cells, mean ± SEM) on K562, K562-CD19 and Raji cells. (B) Histogram representing the frequency of CD20 positive cells (gated on living cells, mean ± SEM) on K562 and Raji cells. Each data point represents one experiment.

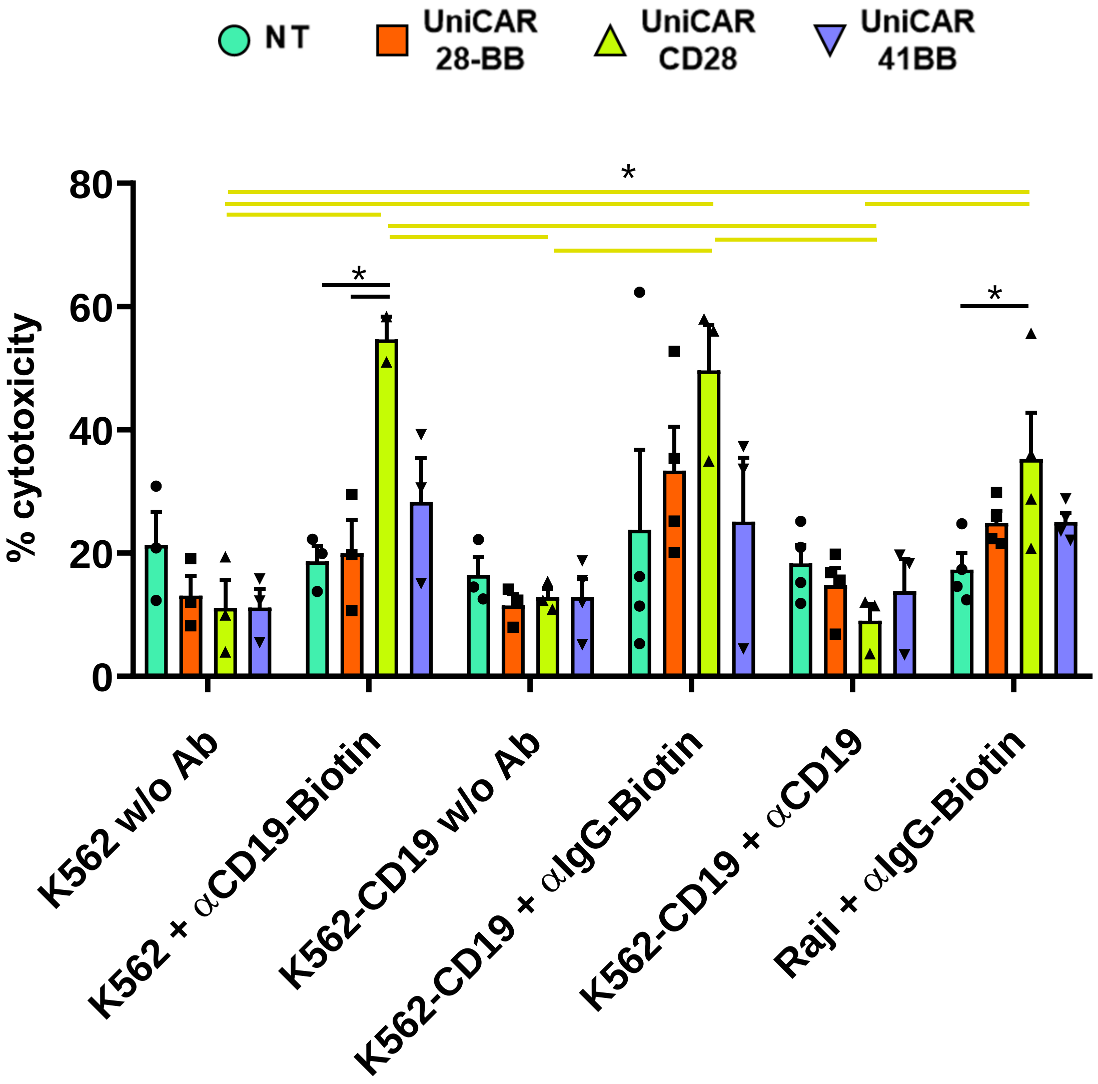


**Supplemental Figure 4: Cytotoxicity assay on cell lines.** *: Significant differences p<0.05, inter-conditions are indicated by colored lines which correspond to the histogram's color code. Intra-conditions are indicated by black lines. Significant differences were determined using a 2way ANOVA followed by Tukey's multiple comparisons test.

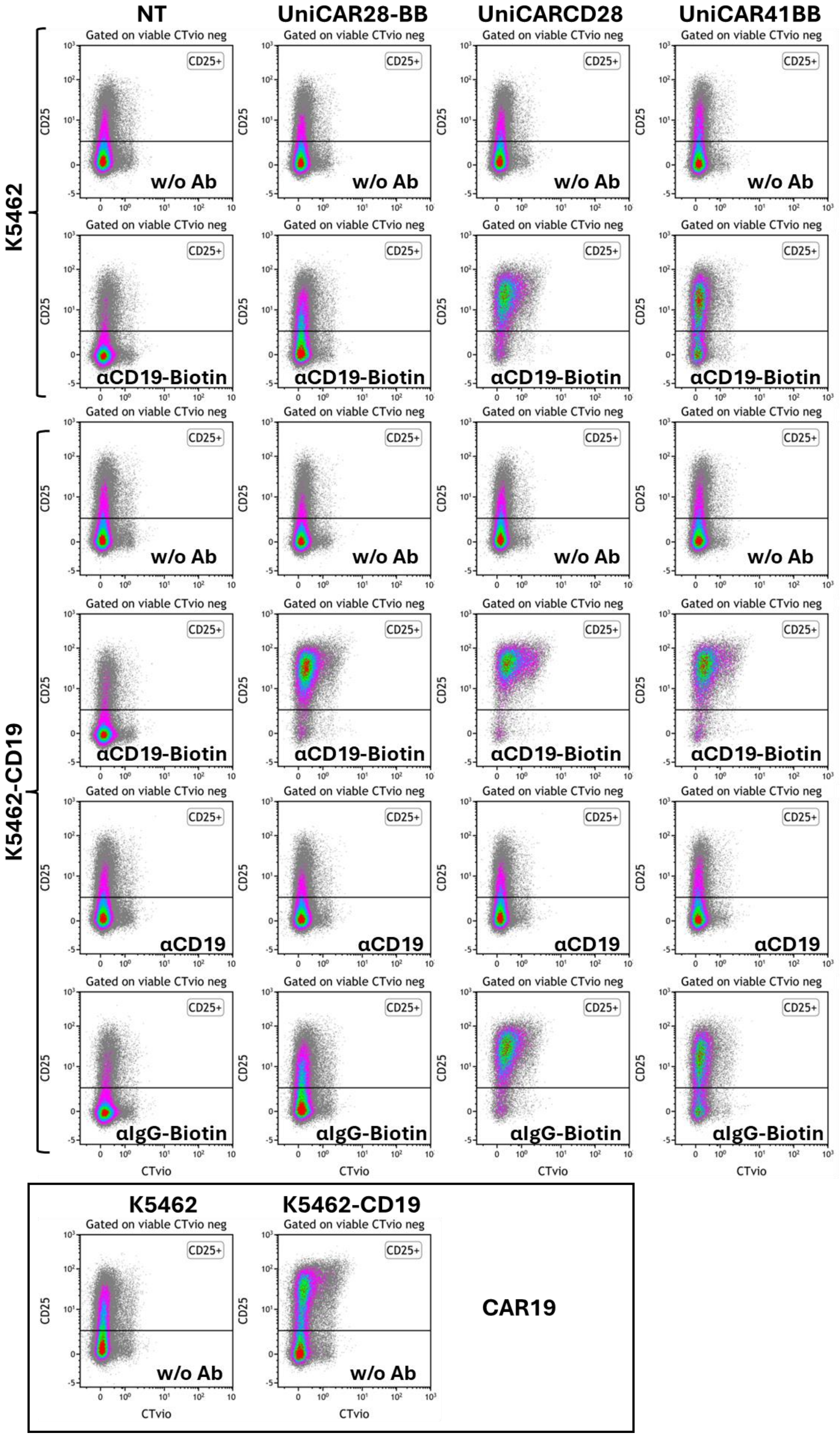
NT
UniCAR28-BB
UniCARCD28
UniCAR41BB
K5462
K5462-CD19
Gated on viable CTvio neg
CD25+
CD25
CTvio
w/o Ab
αCD19-Biotin
αCD19
αIgG-Biotin
K5462
K5462-CD19
CAR19

**Supplemental Figure 5: Expression of CD25 on non-transduced or genetically modified effector T cells in coculture assay.** Dot plot from one independent experiments of all conditions represented in Figure 5A. These dot plots serve as a representative example from N=3 for K562 coculture conditions, and N=6 or K562-CD19 coculture conditions.

A

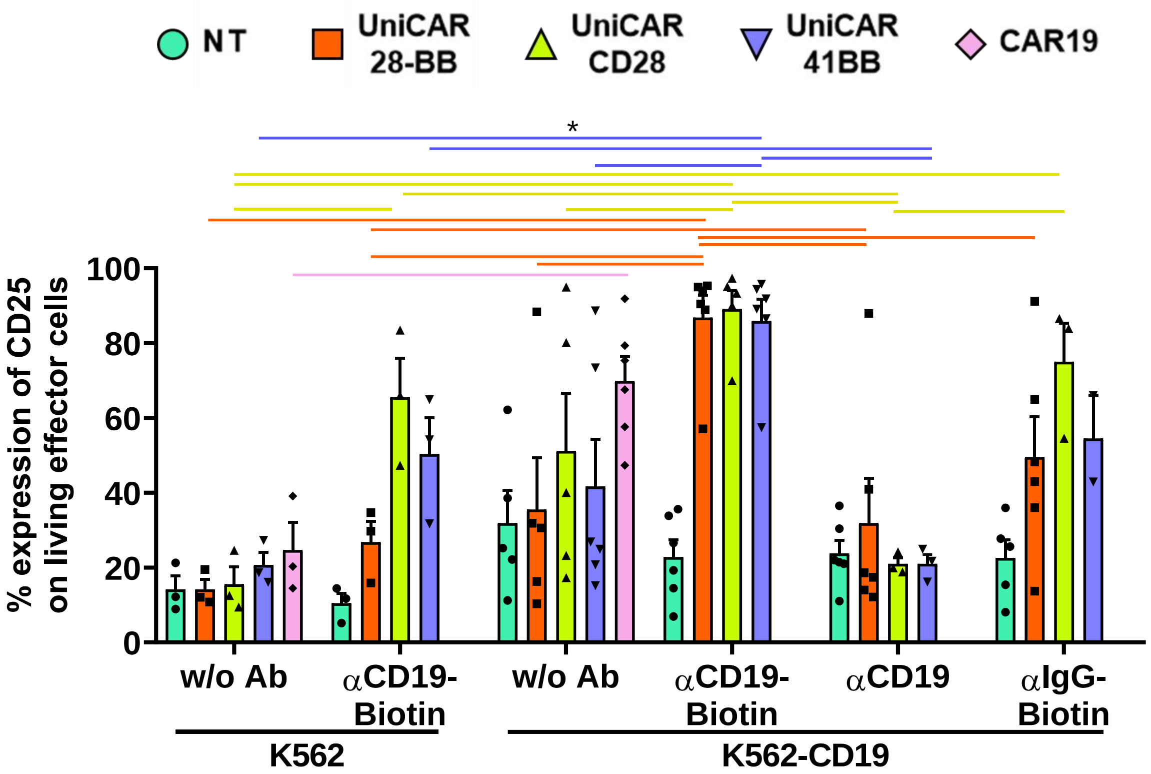
NT
UniCAR 28-BB
UniCAR CD28
UniCAR 41BB
CAR19
*
% expression of CD25 on living effector cells
0
20
40
60
80
100
w/o Ab
αCD19-Biotin
w/o Ab
αCD19-Biotin
αCD19
αIgG-Biotin
K562
K562-CD19

B

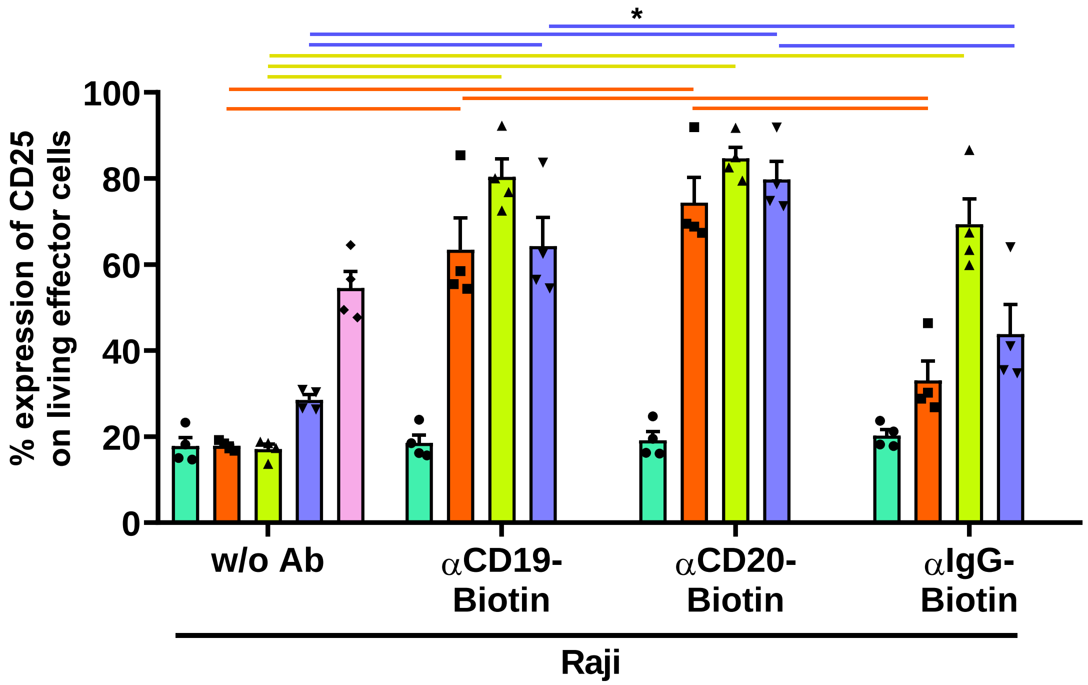
*
% expression of CD25 on living effector cells
0
20
40
60
80
100
w/o Ab
αCD19-Biotin
αCD20-Biotin
αIgG-Biotin
Raji

**C**

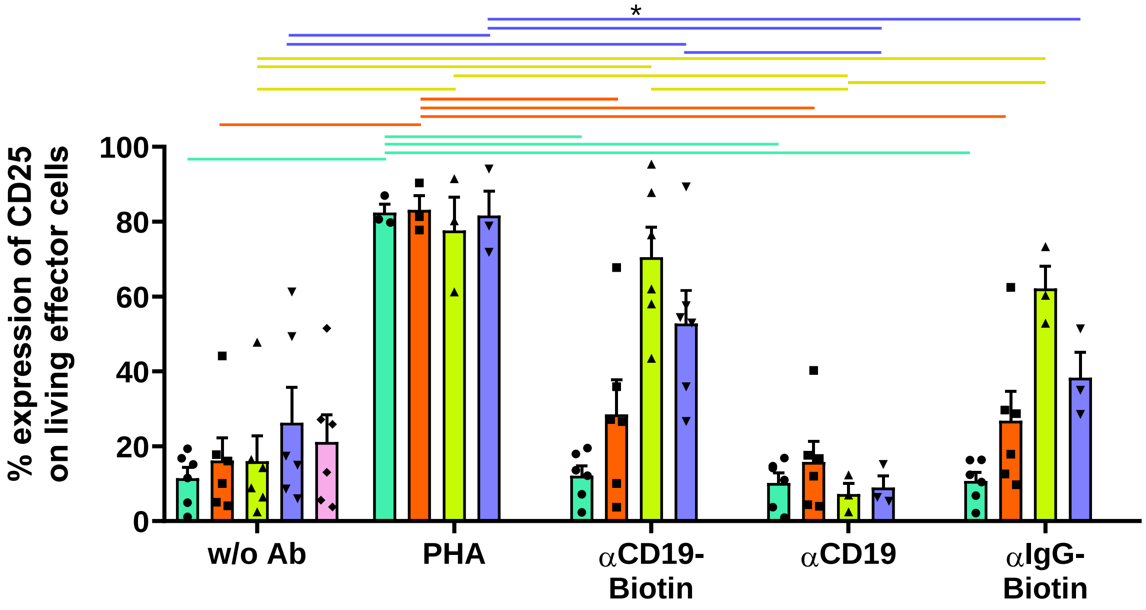


**Supplemental Figure 6: CD25+ effector T cells (Non transduced or genetically modified).** (A) Histogram representing the frequencies of CD25+ cells on NT, UniCAR-28-BB, UniCARCD28, UniCAR41BB and CAR19 T cells (gated on living cells, mean ± SEM) after 72 h of co-culture with CTVio+ K562 and CTVio+ K562-CD19 cells, in the absence of antibody intermediate, or with biotinylated αCD19 Ab, non-biotinylated αCD19 Ab or with biotinylated IgG. (B) Histogram representing the frequencies of CD25+ cells on NT, UniCAR-28-BB, UniCARCD28, UniCAR41BB and CAR19 T cells (gated on living cells, mean ± SEM) after 72 h of co-culture with CTVio+ Raji cells, in the absence of intermediate antibody, or with biotinylated αCD19 Ab, biotinylated αCD20 Ab or with biotinylated IgG. (C) Histogram representing the frequencies of CD25+ cells on NT, UniCAR-28-BB, UniCARCD28, UniCAR41BB and CAR19 T cells (gated on living cells, mean ± SEM) after 72 h of culture alone in the absence of intermediate antibody, or with PHA, biotinylated αCD19 Ab, biotinylated αCD20 Ab or biotinylated IgG. *: Significant differences inter-conditions are indicated by colored lines which correspond to the histogram's color code. Significant differences were determined using a 2way ANOVA followed by Tukey's multiple comparisons test, with significance when $p<0.05$.

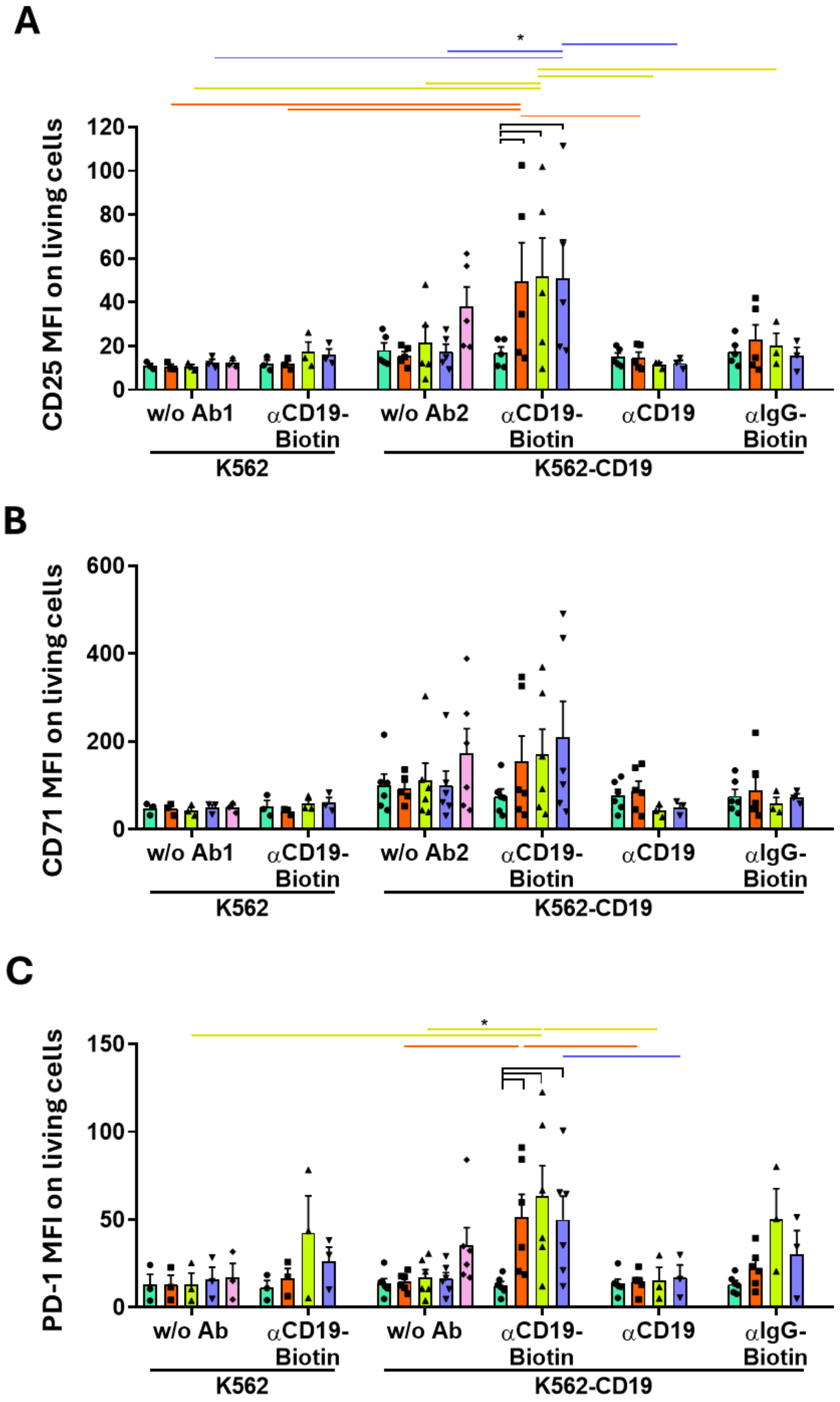
A
*
CD25 MFI on living cells
120
100
80
60
40
20
0
w/o Ab1
αCD19-Biotin
w/o Ab2
αCD19-Biotin
αCD19
αIgG-Biotin
K562
K562-CD19
B
CD71 MFI on living cells
600
400
200
0
w/o Ab1
αCD19-Biotin
w/o Ab2
αCD19-Biotin
αCD19
αIgG-Biotin
K562
K562-CD19
C
*
PD-1 MFI on living cells
150
100
50
0
w/o Ab
αCD19-Biotin
w/o Ab
αCD19-Biotin
αCD19
αIgG-Biotin
K562
K562-CD19

**Supplemental Figure 7: Mean of Fluoresecne Intensity (MFI) of the activation markers CD25, CD71 and PD-1 on effector T cell in co-culture conditions.** (A) Histogram representing the MFI of CD25, (B) of CD71 or (C) PD-1 markers (mean ± SEM, gated on living cells) on NT, UniCAR-28-BB, UniCARCD28, UniCAR41BB and CAR19 T cells after 72 h co-cultured with K562 and K562-CD19, in the absence of intermediate antibody (w/o Ab), or with biotinylated αCD19 Ab (+ αCD19-Biotin), non-biotinylated αCD19 Ab (αCD19), biotinylated αCD20 Ab (+ αCD20-Biotin), or biotinylated non relevant IgG Ab (+ αIgGBiotin). All co-culture conditions were performed with a ratio 1:1 (effector T cell : Target cell). Each data point represents one experiment. *: Significant differences inter-conditions are indicated by colored lines which correspond to the histogram's color code. Black horizontal brackets represent significant differences into a same condition. Significant differences were determined using a 2way ANOVA followed by Tukey's multiple comparisons test, with significance when $p<0.05$.

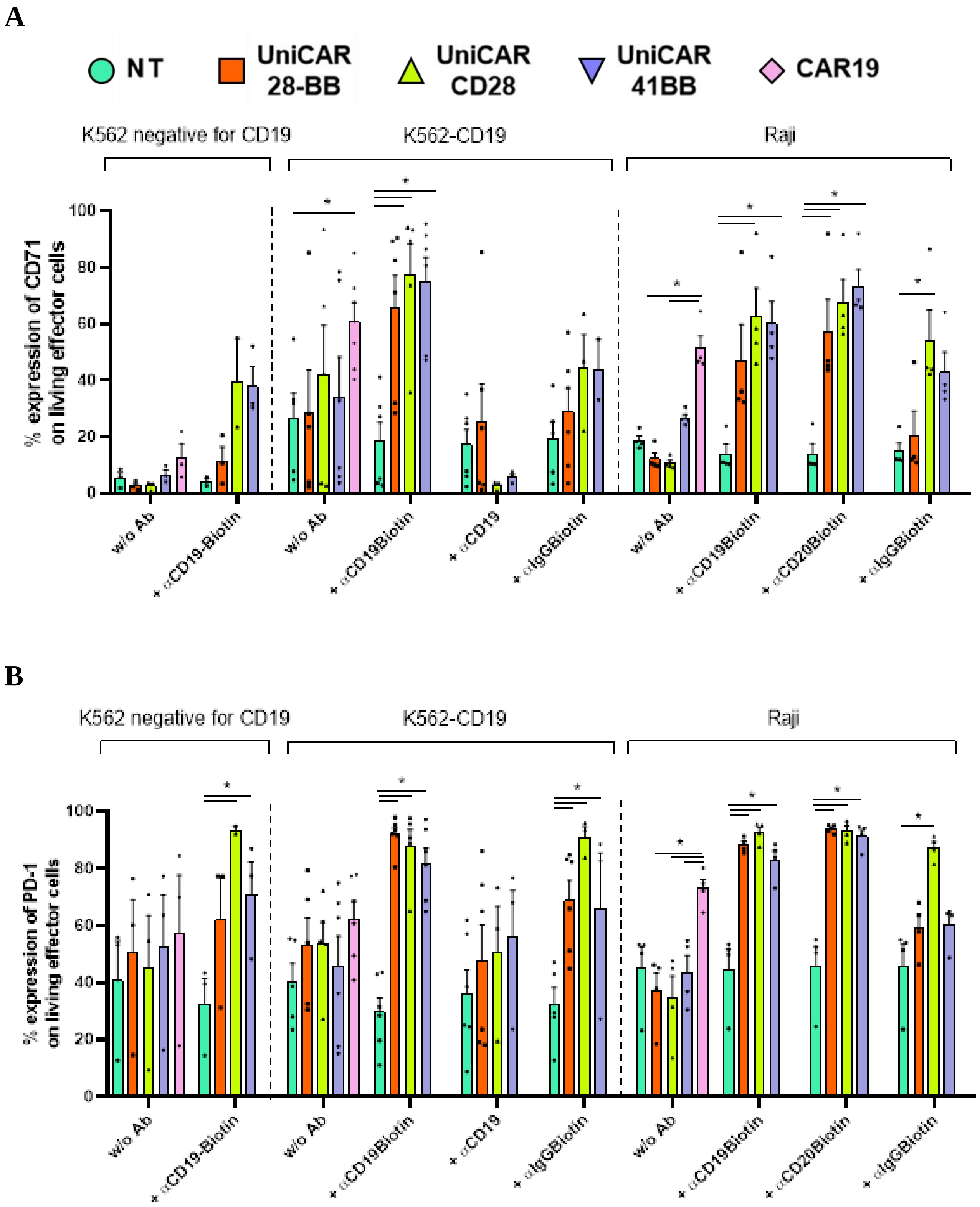
A
NT
UniCAR 28-BB
UniCAR CD28
UniCAR 41BB
CAR19
K562 negative for CD19
K562-CD19
Raji
% expression of CD71 on living effector cells
w/o Ab
+ αCD19-Biotin
w/o Ab
+ αCD19Biotin
+ αCD19
+ αIgGBiotin
w/o Ab
+ αCD19Biotin
+ αCD20Biotin
+ αIgGBiotin
B
K562 negative for CD19
K562-CD19
Raji
% expression of PD-1 on living effector cells
w/o Ab
+ αCD19-Biotin
w/o Ab
+ αCD19Biotin
+ αCD19
+ αIgGBiotin
w/o Ab
+ αCD19Biotin
+ αCD20Biotin
+ αIgGBiotin

**Supplemental Figure 8: Expression of the activation markers CD71 and PD-1 on effector T cell in co-culture conditions.** (A) Histogram representing the frequencies of CD71 or (B) PD-1 markers (mean ± SEM, gated on living cells) on NT, UniCAR-28-BB, UniCARCD28, UniCAR41BB and CAR19 T cells after 72 h co-cultured with K562, K562-CD19 and Raji cells, in the absence of intermediate antibody (w/o Ab), or with biotinylated αCD19 Ab (+ αCD19-Biotin), non-biotinylated αCD19 Ab (αCD19), biotinylated αCD20 Ab (+ αCD20-Biotin), or biotinylated non relevant IgG Ab (+ αIgGBiotin). All co-culture conditions were performed with a ratio 1:1 (effector T cell : Target cell). Each data point represents one experiment. *: Significant differences between conditions were determined using a 2way ANOVA followed by Tukey's multiple comparisons test, with significance when $p<0.05$.

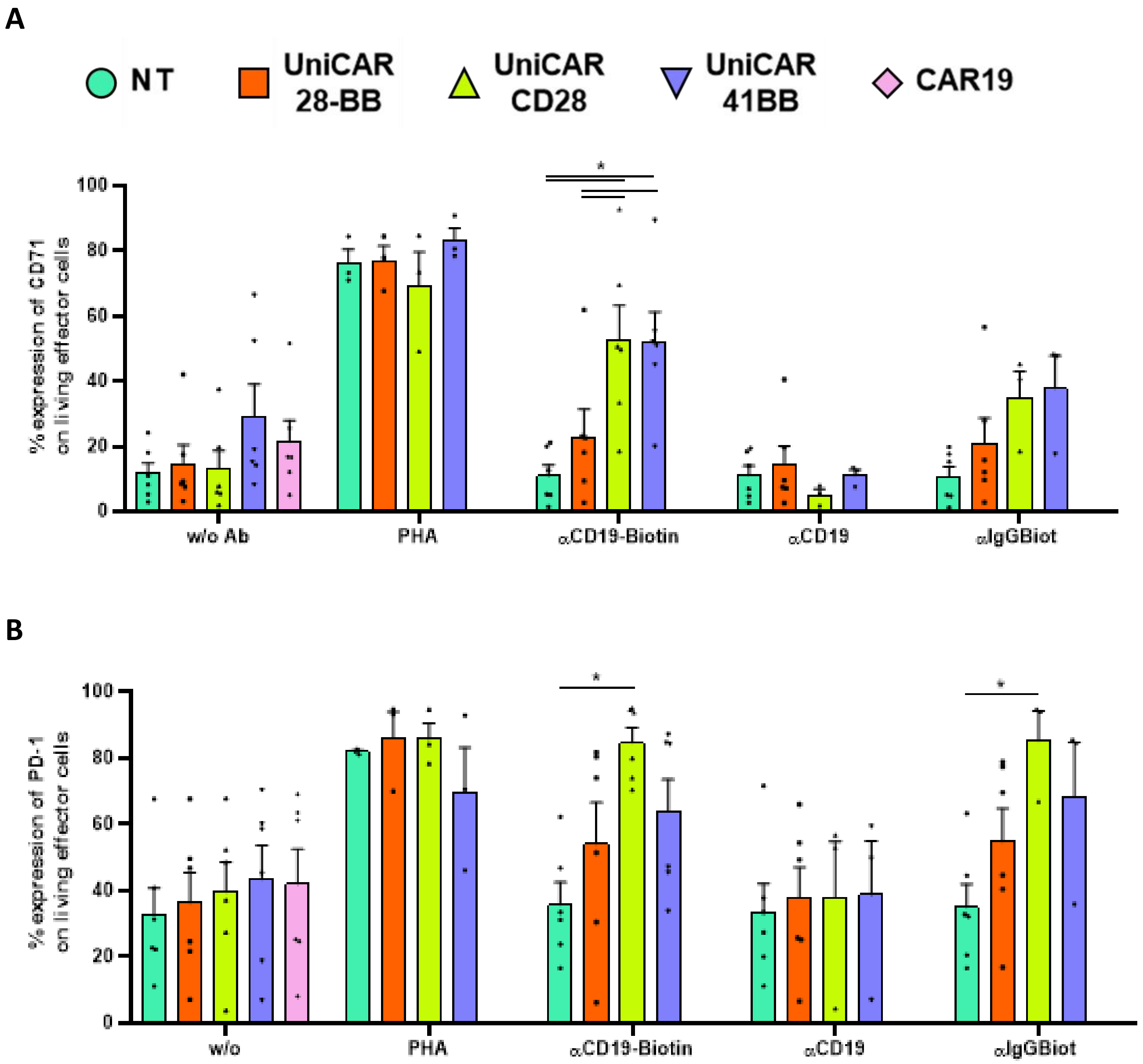


**Supplemental Figure 9: Expression of the activation markers CD71 and PD-1 on effector T cell, cultured alone.** (A) Histogram representing the frequencies of CD71 or (B) PD-1 markers (mean ± SEM, gated on living cells) on NT, UniCAR-28-BB, UniCARCD28, UniCAR41BB and CAR19 T cells alone, without coculture, 72 h after stimulation without Ab (w/o Ab), or with PHA, biotinylated αCD19 Ab (+ αCD19-Biotin), non-biotinylated αCD19 Ab (αCD19), or biotinylated non relevant IgG Ab (+ αIgGBiotin). Each data point represents one experiment. *: Significant differences between conditions were determined using a 2way ANOVA followed by Tukey's multiple comparisons test, with significance when $p<0.05$.
